# Key sub-community dynamics of medium-chain carboxylate production

**DOI:** 10.1186/s12934-019-1143-8

**Published:** 2019-05-28

**Authors:** Johannes Lambrecht, Nicolas Cichocki, Florian Schattenberg, Sabine Kleinsteuber, Hauke Harms, Susann Müller, Heike Sträuber

**Affiliations:** Department of Environmental Microbiology, Helmholtz Centre for Environmental Research, Permoserstr. 15, 04318 Leipzig, Germany

**Keywords:** Single cell analytics, Microbial chain elongation, Microbial community, Flow cytometry, 16S rRNA gene sequencing, Process monitoring, Caproic acid, Caprylic acid, MCFA, Anaerobic fermentation

## Abstract

**Background:**

The carboxylate platform is a promising technology for substituting petrochemicals in the provision of specific platform chemicals and liquid fuels. It includes the chain elongation process that exploits reverse β–oxidation to elongate short-chain fatty acids and forms the more valuable medium-chain variants. The pH value influences this process through multiple mechanisms and is central to effective product formation. Its influence on the microbiome dynamics was investigated during anaerobic fermentation of maize silage by combining flow cytometric short interval monitoring, cell sorting and 16S rRNA gene amplicon sequencing.

**Results:**

Caproate and caprylate titres of up to 6.12 g L^−1^ and 1.83 g L^−1^, respectively, were achieved in a continuous stirred-tank reactor operated for 241 days. Caproate production was optimal at pH 5.5 and connected to lactate-based chain elongation, while caprylate production was optimal at pH 6.25 and linked to ethanol utilisation. Flow cytometry recorded 31 sub-communities with cell abundances varying over 89 time points. It revealed a highly dynamic community, whereas the sequencing analysis displayed a mostly unchanged core community. Eight key sub-communities were linked to caproate or caprylate production (r_S_ > | ± 0.7|). Amongst other insights, sorting and subsequently sequencing these sub-communities revealed the central role of *Bifidobacterium* and *Olsenella*, two genera of lactic acid bacteria that drove chain elongation by providing additional lactate, serving as electron donor.

**Conclusions:**

High-titre medium-chain fatty acid production in a well-established reactor design is possible using complex substrate without the addition of external electron donors. This will greatly ease scaling and profitable implementation of the process. The pH value influenced the substrate utilisation and product spectrum by shaping the microbial community. Flow cytometric single cell analysis enabled fast, short interval analysis of this community and was coupled with 16S rRNA gene amplicon sequencing to reveal the major role of lactate-producing bacteria.

**Electronic supplementary material:**

The online version of this article (10.1186/s12934-019-1143-8) contains supplementary material, which is available to authorized users.

## Background

To date the provision of platform chemicals and liquid fuels is still predominantly based on fossil resources. These petrochemicals need to be substituted by biomass conversions to facilitate a circular bio-economy that can sustainably satisfy the needs of a growing world population. A comparison of different technologies available for this task has shown the highest overall yields for the carboxylate platform [1]. The carboxylate platform is based on short-chain fatty acids produced by anaerobic fermentation being elongated to medium-chain fatty acids (MCFAs) [2] by reverse β-oxidation [3, 4]. This chain elongation (CE) process is characterised by the stepwise addition of acetyl groups to carboxylates. These C2 groups can be provided by ethanol [5, 6] or lactate [4, 7–10]. In the following, carboxylic acids are referred to as carboxylates (e.g., acetate—C2, butyrate—C4, caproate—C6 and caprylate—C8) and designate the unbranched compounds if not specified differently.

The C6 and C8 carboxylates (caproate and caprylate) are valuable CE products with promising applications, e.g. as feed additives [11], antimicrobial agents [12], and corrosion inhibitors [13]. Furthermore, they can act as building blocks for fuels [14], organic solvents [3], and bioplastics [15] as well as precursors for flavours and fragrances [16]. Even-numbered MCFAs are mainly produced from palm kernel and coconut oil [17], which is connected to serious socio-economic and ecological problems [18], whereas odd-numbered carboxylates are still produced from fossil resources. In contrast, biotechnological MCFA production relies on microbial communities fermenting a wide spectrum of readily available biomass and organic waste streams [19].

The ability to produce MCFAs seems to be widespread among microorganisms in anaerobic fermentation communities, but the accomplishable yields are limited by product inhibition [20]. The pH value in the fermentation broth is a major parameter controlling this inhibition. It influences the process through multiple mechanisms. An acidic pH value around 5.5 increases product toxicity and generally decreases hydrolytic performance [21], but in turn reliably suppresses methanogenesis [22] and facilitates potential product separation approaches, such as extraction [11]. Conversely, a neutral pH around 7 reduces product toxicity and increases hydrolytic and fermentation performance [23], but might facilitate methanogenesis and complicate potential extraction approaches. The increased product toxicity at lower pH values is induced by a higher share of undissociated and therefore uncharged carboxylic acids (pK_a_ values: acetate 4.76, butyrate 4.82, caproate 4.85, caprylate 4.89). Undissociated carboxylic acids can pass cell membranes and disrupt the membrane structure [24]. Within the cell, they dissociate and thereby acidify the cytoplasm, which is usually of neutral pH [25]. Dissociated acids accumulate in the cell and induce further cytotoxic effects by disrupting the amino acid production [26]. A controlled increase in environmental pH should minimise the share of undissociated carboxylic acids, thereby reducing diffusion and product toxicity and ultimately increasing carboxylate production. However, at the same time increased pH values might progressively promote methanogenesis, a potentially competing and very effective pathway of biomass conversion as methane contains the lowest free energy content per electron under anaerobic conditions [11].

A wide variety of organisms has been associated with anaerobic fermentation and CE processes [11]. Model strains for CE, such as *Clostridium kluyveri* and *Megasphaera elsdenii*, have been described and intensively investigated in pure culture [19]. However, these species are mostly not detected in chain elongating microbial communities [8, 27], while the functions of other taxa present in these communities are still not comprehensively understood.

Here, we applied flow cytometric single cell analysis to elucidate the structure and dynamics of carboxylate-producing microbial communities. This high through-put technique allows high frequency routine monitoring of complex communities [28–30] and has recently been applied to examine cell activity states in a MCFA-producing microbial community [27]. Microbial flow cytometry has been proven to provide valuable structure–function relationships in anaerobic lab-scale [31] and full-scale systems [32] by implementing correlation analyses and biostatistics tools.

In this study, we investigated the microbial MCFA production process at different pH conditions. Specifically, we aimed to elucidate the effects of the pH value on community dynamics, the selective substrate utilisation and the resulting product spectrum. Maize silage was used as model substrate containing lactate and ethanol. We hypothesise that the microbial community structure is interlinked with CE efficiency and that key sub-communities may emerge depending on substrate usage and pH variations. Cell sorting and Illumina MiSeq 16S rRNA gene amplicon sequencing were applied to verify cytometric measurements and the resulting correlation analyses.

## Results

### Process performance

**Fig. 1:**
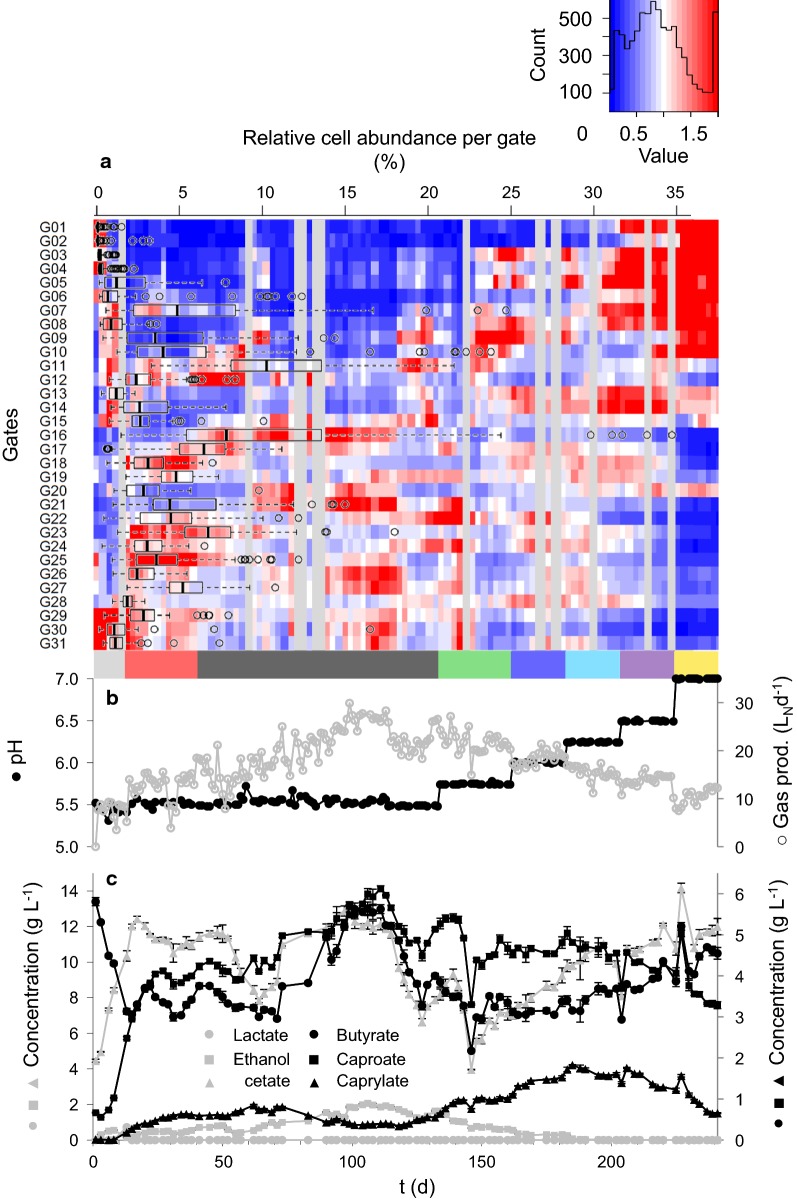
Compilation of the microbial community dynamics along the eight experimental stages in **a** and the process parameters in **b** and **c** over the course of 241 days. **a** A flowCyBar representing the frequency distribution and the development of the relative abundances of all 31 sub-communities (gating strategy in Additional file 1: S8). Blue fields mark negative and red ones positive deviations from the mean relative abundance of the respective sub-community. The duration of the experimental stages is colour-coded: 1—start-up  , 2—TAN-shortage  , 3—consolidation  , 4—pH 5.75  , 5—pH 6.0  , 6—pH 6.25  , 7—pH 6.5  and 8—pH 7  . **b** Shows the pH value  and the gas production  . **c** Shows the concentrations of the major compounds involved in the chain elongation process: lactate  , ethanol  , acetate  , butyrate ●, caproate ■ and caprylate ▲

A 15 L reactor set-up was designed to promote anaerobic fermentation and CE for microbial MCFA production. The reactor was operated for 241 days with a hydraulic retention time (HRT) of 4 days. The reactor pH value was controlled automatically and kept at pH 5.5 until day 134. Then it was increased stepwise until pH 7.0 was reached after 225 days (Fig. 1). The whole experiment was divided into eight stages: stage 1—start of the reactor and adaptation of the microbial community until day 12, stage 2—total ammonia nitrogen (TAN) shortage until day 40, stage 3—consolidation of the system until day 133, stage 4—pH 5.75 (day 134–161), stage 5—pH 6.0 (day 162–182), stage 6—pH 6.25 (day 183–203), stage 7—pH 6.5 (day 204–224), and stage 8—pH 7.0 (day 225–241). Stages 4 to 7 lasted 21 days each (equalling 5.25 × HRT each), whereas stage 8 at pH 7.0 was stopped after 17 days (equalling 4.25 × HRT) due to increasing methane production (Fig. 1, Additional file 1: S4).

On average 26.9% ± 8.5% of the volatile solids (VS) in the substrate were degraded over the whole experiment. The degree of VS degradation in the eight experimental stages ranged from 20.9% ± 7.2% during the start-up phase to 32% ± 6.5% at pH 7.0 (Additional file 1: S3).

An initial period of increasing caproate and caprylate concentrations started after 1.5 HRTs on day 6 during stage 1 and lasted until day 45 with product concentrations of 4.36 g L^−1^ and 0.6 g L^−1^, respectively. The concentrations of caproate and caprylate reached their maxima on day 111 during stage 3 (pH 5.5, 6.12 g L^−1^ caproate) and on day 185 during stage 6 (pH 6.25, 1.83 g L^−1^ caprylate), respectively. The production efficiencies were 2.04 g L^−1^ day^−1^ for caproate and 0.54 g L^−1^ day^−1^ for caprylate. Both ethanol and lactate can serve as electron donor for CE. Lactate was added to the fermenter at high concentrations with the daily feed (up to 74.68 g kg_TS_^−1^) but was not detected in the fermentation broth except on day 10 (0.227 g L^−1^) and day 13 (0.721 g L^−1^). The data suggest that lactate was effectively used for CE during all stages of the experiment, irrespective of the changing pH values. The ethanol concentration displayed different trends and increased until day 106 (2.07 g L^−1^). It subsequently decreased to zero until stage 6 (day 199, Fig. 1) while the caprylate concentration increased simultaneously. Therefore, it can be assumed that CE with ethanol might be connected to the increased caprylate production starting in stage 4. However, caproate and caprylate concentrations generally decreased with pH values close to neutral in stages 7 and 8. The results suggest pH optima for caproate formation at 5.5 and pH optima for caprylate production between 6.0 and 6.25.

Propionate and longer-chained odd-numbered carboxylates only accumulated in considerable quantities at the end of stage 1 (day 8, 2.53 g L^−1^ propionate) and stage 8 (day 241, 1.79 g L^−1^ propionate, Additional file 1: S7). Propionate concentrations were substantially lower during all other stages and reached a minimum on day 146 (pH 5.75, 0.07 g L^−1^ propionate).

With 12.5% CH_4_ in the headspace and a methane yield of 4.38 mL CH_4_ per g VS on day 225, and even 23.6% CH_4_ and 12.66 mL CH_4_ per g VS on day 241 we found considerable methanogenic activity during stage 8 (pH 7.0). In the stages 1 to 6, methane production was inhibited by the combination of acidic pH and additional factors, such as low HRT and a comparably high salinity. The methane concentrations in the gas were below 1.1% in the stages 1–3 at pH 5.5, below 2.3% in stage 4 at pH 5.75, below 3.1% in stage 5 at pH 6.0 and below 4.2% in stage 6 at pH 6.25. The daily gas production increased continuously from stage 1 (6.13 L_N_ day^−1^ to 9.31 L_N_ day^−1^) to stage 3 (29.91 L_N_ day^−1^, day 99) and decreased again until the end of the experiment (12.24 L_N_ day^−1^). While the major gas component was CO_2_ (Additional file 1: S4), the hydrogen concentration stabilised at around 30% in stage 2 and started to decrease continuously at the start of stage 4 to reach values below 5% towards the end of the experiment. A noticeable drop in the hydrogen concentration at the beginning of stage 5 (day 164, pH 6) coincided with the first slight increase in methane production and might hint towards hydrogenotrophic methanogenesis.

### Microbial community dynamics

**Fig. 2:**
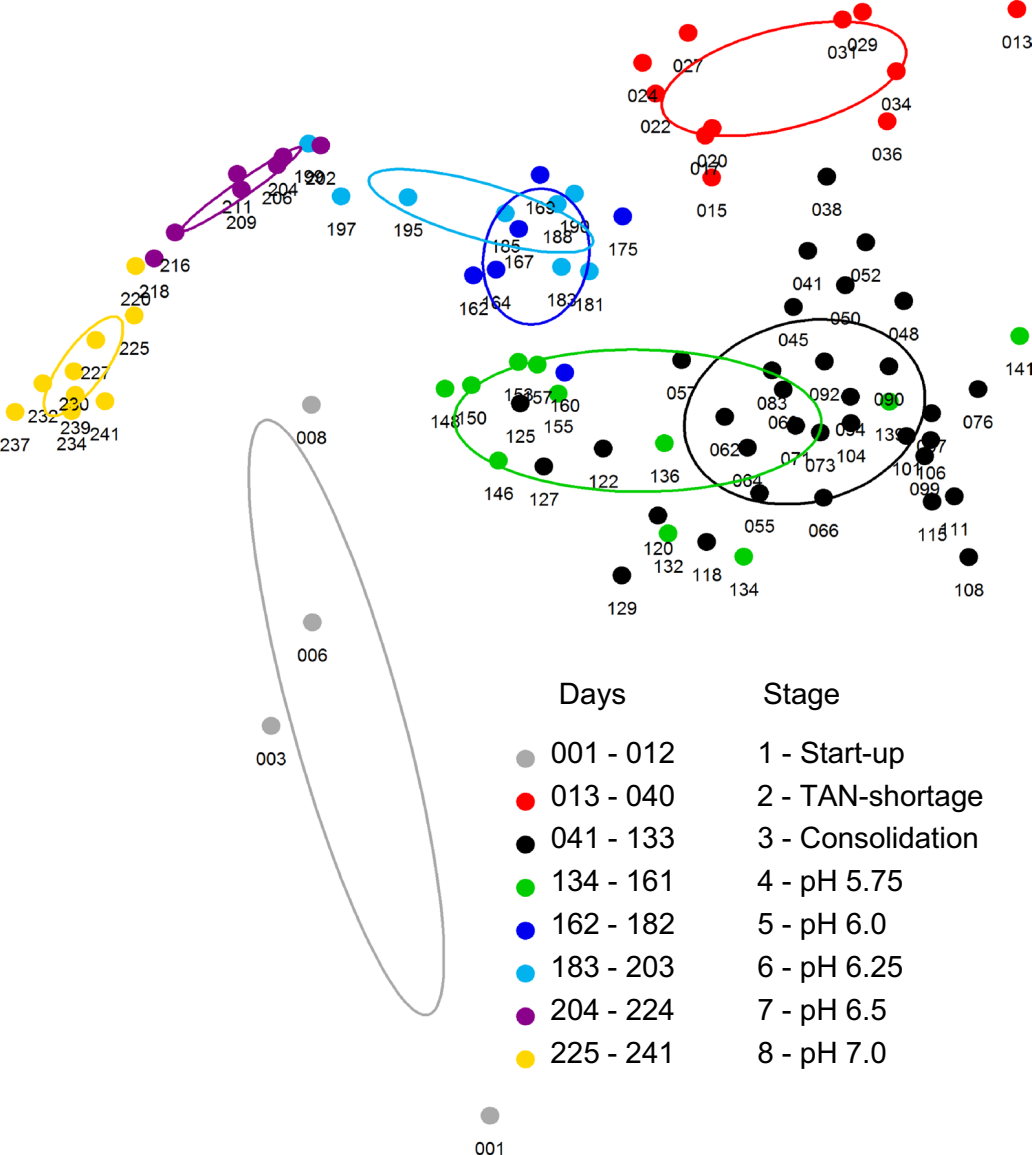
flowCHIC analysis of the 89 cytometric fingerprints revealed clustering of similar microbial communities over time in an NMDS plot. The sampling points are assigned to their corresponding experimental stages. The dispersion ellipses mark the standard deviation of samples within the specific groups around the weighted average of the group. The NMDS plot is based on a stress value of 0.155

A total of 89 sample time points were analysed to realise an average sampling interval below the HRT. The cytometric fingerprints were recorded in forward scatter (FSC) vs. DAPI (4′,6-diamidin-2-phenylindol) fluorescence dot plots and evaluated using a master gate template with 31 sub-communities (Additional file 1: S8) [33]. An animated sequence of these fingerprints (Additional file 2) illustrates the variations in community structures over the 241 days of reactor operation. The community structure was routinely measured in all stages. Its dynamics were visualised with the flowCHIC tool and are shown in Fig. 2. The non-metric multidimensional scaling (NMDS) plot generated from the cytometric data revealed a clear dependence between the community structure and the eight experimental stages (Fig. 2). The start-up period was characterised by severe day to day changes that resulted in distinct community structures during the TAN-shortage in stage 2 and the consolidation in stage 3. The community was similar in stage 4 but the structure changed with subsequent pH increases and distinctly shifted away from the consolidation stage. The microbiome dynamics were further resolved to sub-communities and visualised with the flowCyBar tool (Fig. 1a, relative cell abundances listed in Additional file 1: S10) using the relative cell abundances in 31 gates (G01 to G31, Additional file 1: S8, S10). Each sample is colour-coded from dark blue to dark red for below to above average relative cell abundance of a specific gate. The average abundance is coded in white and differs greatly between the gates (min. 0.087% in G01 to max. 11.042% in G11; Additional file 1: S10). The box plot overlay in Fig. 1a highlights this uneven relative cell abundance distribution between the sub-communities.

The flowCyBar plot confirmed the initial adaptations of the community to reactor conditions in stage 1 and revealed long term trends of relative cell abundances in the sub-communities G01 to G10 concurrently to the pH gradient in the stages 4 to 8. In contrast, decreasing relative cell abundances were displayed in G16 to G19 and G21 to G31 at the beginning of stage 8 (pH 7.0). However, some sub-communities did not show such general trends but short-term shifts in relative cell abundances per gate.

### Sub-communities correlating to caproate and caprylate production

**Fig. 3:**
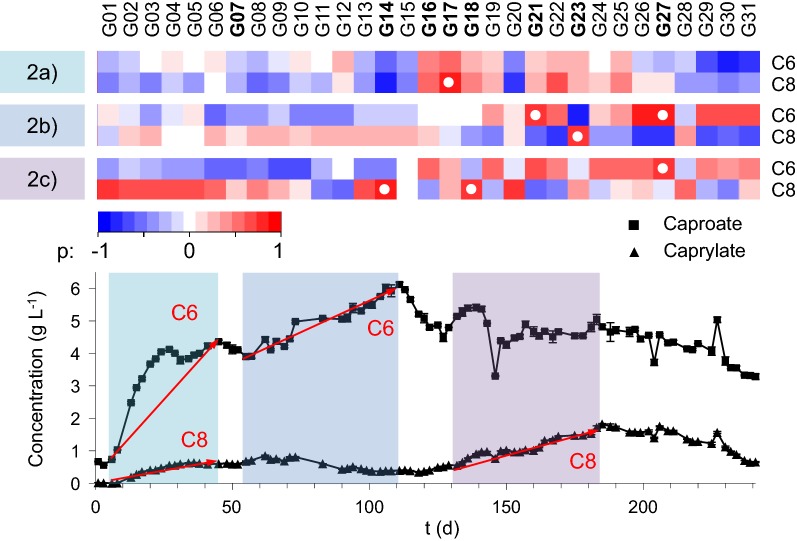
Spearman’s rank order correlation was applied to test for links between the relative cell abundance values of all 31 sub-communities and caproate (C6) and caprylate (C8) concentrations. The correlation approach comprised the three periods: (2a) the initial caproate and caprylate increase from day 6 to day 45  , (2b) the second caproate increase from day 57 to day 111  and (2c) the second caprylate increase from day 132 to day 185  were analysed. Correlation strength (r_S_), significance (p) and corrected significance (p_BH_) are provided in Additional file 1: Table 1 S11. Sub-communities chosen for sorting are marked in bold and additionally provided in Additional file 1: Table 3 S11. The strong correlations (r_S_ > | ± 0.7|) these sub-communities were chosen for are marked with a white dot

The changes in relative cell abundance in all 31 gates were correlated to caproate and caprylate concentrations to identify key sub-communities relevant for product formation. For this, data were divided into subsets according to two approaches (1) the experimental stages (Additional file 1: S11) and (2) the periods of major product concentration increase (Fig. 3) to identify key sub-communities linked to CE. Both approaches applied Spearman’s rank order coefficient r_S_ to test the correlations according to their strength (coefficient r_S_, threshold > | ± 0.7|) and corrected significance (p_BH_ threshold < 0.05). Sub-communities displaying strong, significant correlations in both analyses were further evaluated by their relative cell abundance values and trends. A 5% relative cell abundance threshold was applied to determine key sub-communities related to process performance. Positive correlations between gates and target carboxylate concentration were focused and interpreted as indications for CE supporting organisms. The analysis also included one notably abundant negatively correlated sub-community to unveil potential CE antagonists or MCFA-degrading bacteria.

The first correlation analysis comprised the three periods: (1a) stage 2, (1b) stage 3 and (1c) stages 4 to 7. In (1a) G17 correlated strongly positive (r_S_ = 0.73, p_BH_ = 0.053) with the caprylate concentration (Additional file 1: S11). In (1b) G21 (r_S_ = 0.73, p_BH_ = 1.3 × 10^−5^) and G27 (r_S_ = 0.74, p_BH_ = 6.7 × 10^−8^) showed strong positive correlations to caproate titres, a behaviour also found in (1c) (G21 r_S_ = 0.73, p_BH_ = 1.1 × 10^−5^, G27 r_S_ = 0.81, p_BH_ = 2.6 × 10^−7^). In contrast, G07 showed strong negative correlation (r_S_ = − 0.7, p_BH_ = 4.5 × 10^−5^) to the caproate concentration in (1c) and G18 correlated strongly positive (r_S_ = 0.8, p_BH_ = 8.2 × 10^−6^) with the caprylate concentration.

The second correlation approach comprised the three periods: (2a) initial increase in caproate and caprylate concentrations from day 6 to 45, (2b) second caproate concentration increase from day 55 to 111 and (2c) second caprylate concentration increase from day 132 to 185. In (2a) G17 correlated positively to the caprylate concentration (r_S_ = 0.8, p_BH_ = 0.001). In (2c) G21 and G27 correlated strongly positive (G21 r_S_ = 0.73, p_BH_ = 0.003, G27 r_S_ = 0.81, p_BH_ = 1.4 × 10^−4^) with the caproate concentrations as before. Additionally G23 showed strong positive correlation to caprylate concentrations (r_S_ = 0.79, p_BH_ = 0.001). In (2c) G27 alone correlated strongly positive to the caproate concentrations (r_S_ = 0.8, p_BH_ = 3.9 × 10^−4^) while strong positive correlation of G14 (r_S_ = 0.83, p_BH_ = 1.2 × 10^−4^) and G18 (r_S_ = 0.78, p_BH_ = 0.001) with caprylate concentrations were found.

These findings strongly suggest that the sub-communities G17, G18, G21 and G27 play a role in caproate and/or caprylate formation as they display strong correlations in both analyses. They were consequently chosen for more detailed investigation by cell sorting. Additional sub-communities met the set criteria for at least one correlation scenario. G14 showed relative cell abundances below the 5% threshold during major periods of the experiment but stood out with higher abundances during times of maximum caprylate concentration. G23 reached its maximum relative cell abundances around the time points of peak caproate and caprylate concentrations (days 106 and 185). It correlated negatively to caproate and positively to caprylate, which may hint towards CE from caproate to caprylate. G16 only just failed the selection criterion for correlation strength (r_Scaproate_ = 0.69) but was sorted anyway because it displayed an extraordinarily high relative cell abundance of up to 34% (day 83). G07 was chosen due to negative product correlations, high relative cell abundances during start-up (10.8% at day 8 and 11) and distinct increases in relative abundance at higher pH (4.57% at the beginning of pH 6.25 and up to 24.5% at pH 7.0).

Two time points for each of these eight identified sub-communities were selected for cell sorting. These time points were chosen according to maximum relative cell abundances, maximum product concentrations or time points already chosen for the whole community composition analyses (Additional file 1: S12).

### Community and sub-community composition

**Fig. 4:**
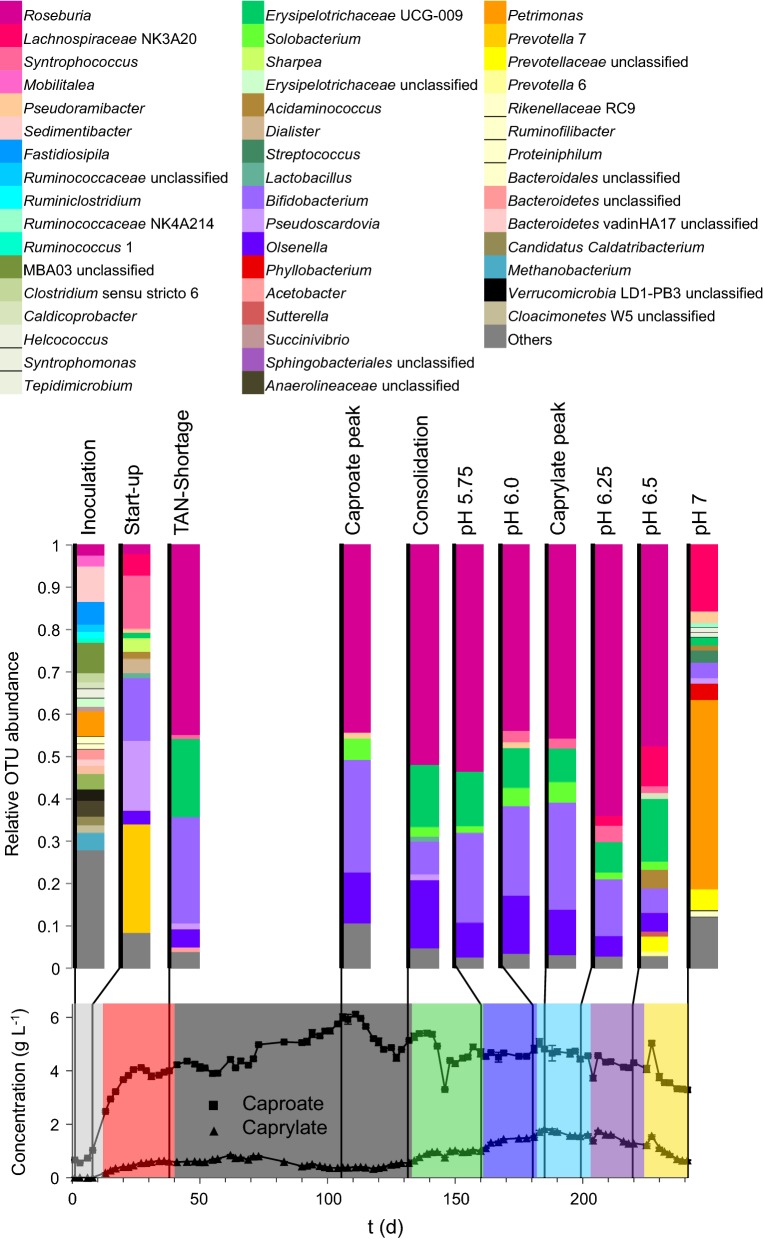
Whole community based on 16S rRNA gene amplicon sequencing at eleven time points along the experiment (day 1, 8, 38, 106, 132, 160, 181, 185, 202, 220, 241). The relative OUT abundances are assigned to time points with the respective caproate ■ and caprylate ▲ concentrations and the eight experimental stages (see Fig. 1). The taxonomic composition is resolved to the genus level applying an abundance threshold of 0.1%. OTUs with abundances below 1% are summarised to “Others”. Details about library preparation, sequencing and sequence data analysis are given in Additional file 1: S13

We investigated the whole community by 16S rRNA gene amplicon sequencing at eleven time points to verify the cytometric data and affiliate the cytometric fingerprints to taxonomic community compositions (Fig. 4). The sequencing results confirmed the pronounced initial community shift during stages 1 and 2 that was revealed by the cytometric approach. This adaptation to the reactor conditions resulted in a core microbial community that was taxonomically stable in composition until the end of stage 7 (day 220). In contrast, the cytometric data displayed substantial variations in community structure during this period (Fig. 2). The sequencing approach revealed a second shift in taxonomic community composition after the pH increase to 7.0 in stage 8 (Fig. 4).

The inoculum contained considerably more operational taxonomic units (OTUs) than all other samples (26 different OTUs with OTU abundances above 0.1%, a mean OTU abundance of only 2.7% and 27.9% of OTUs with less than 0.1% OTU abundance). Towards the end of stage 1 (day 8) the community already was much less diverse, and *Prevotella_7* reached a relative OTU abundance of over 25%. Four key genera started to establish in the core community at the end of stage 2 and subsequently remained largely stable until stage 7 (*Roseburia* 44.8%–64%, *Erysipelotrichaceae* UCG-009 7.2%–18.5%, *Bifidobacterium* 5.8%–25%, *Olsenella* 4.3%–16.1%), even though process parameters and fermenter performance altered considerably during this period. More different taxa were detected again at higher pH values in stages 7 and 8 (12 and 15 different OTUs > 0.1% on day 220 and day 241, respectively), and several new genera established in the community. *Lachnospiraceae* NK3A20 increased successively and the previous core community was replaced nearly completely. Simultaneously, *Petrimonas* displayed by far the highest OTU abundance in stage 8.

**Fig. 5:**
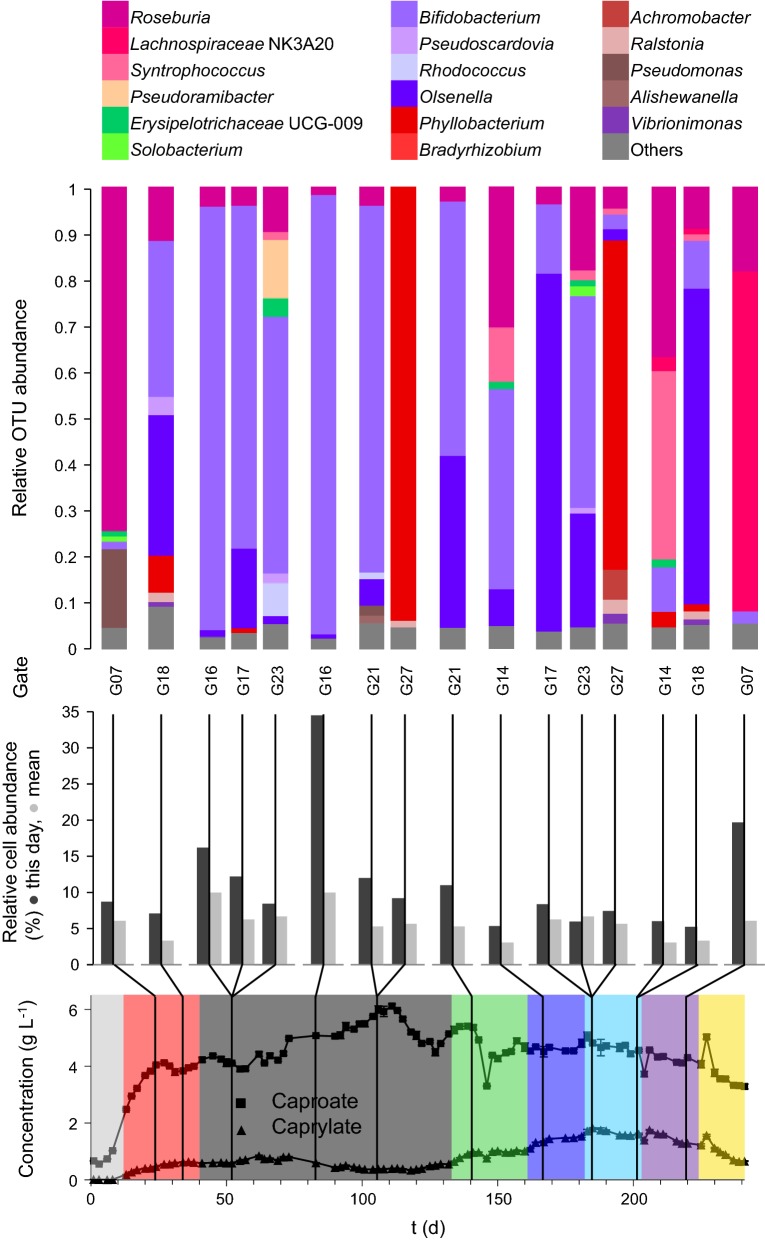
Taxonomic composition of eight sub-communities sorted at different time points due to their strong correlations with the target carboxylates (Fig. 3). Every sub-community composition is given with its relative cell abundance ● and the sub-communities mean relative cell abundance  . The detailed relative cell abundance development is given in Additional file 1: S12. The relative OTU and cell abundances are assigned to time points with the respective caproate ■ and caprylate ▲ concentrations and eight experimental stages (see Fig. 1). The taxonomic composition is resolved to the genus level applying an abundance threshold of 0.1%. OTUs with abundances below 1% are summarised to “Others”. Details about library preparation, sequencing and sequence data analysis are given in Additional file 1: S13

The eight sub-communities G07, G14, G16, G17, G18, G21, G27, G23 strongly correlating to caproate and caprylate titres (Fig. 3) were sorted on two different time points each to reveal key organisms shaping the process (Additional file 1: S12). The sorted sub-communities were analysed for taxonomic composition by Illumina Miseq sequencing of 16S rRNA gene amplicons (Additional file 1: S13). Seven of the sorted sub-communities (G14, G16, G17, G18, G21, G27, G23) together reached a maximum relative cell abundance of 70.28% on day 94 (Fig. 5) and displayed significantly lower relative cell abundances at the beginning and end of the experiment (16.9% in the inoculum, 12% in stage 8 on day 241). The cell sorting partitioned these samples to a degree where some gates effectively contained a single genus (94.8% *Bifidobacterium* in G16 on day 83, 93.8% *Phyllobacterium* in G27 on day 106, Fig. 5). The sorted sub-communities generally displayed a similar composition at the two chosen sampling times. The negatively correlating G07 is a notable exception, as its phylogenetic affiliation shifted from *Roseburia* (74.4% on day 24) to the *Lachnospiraceae* NK3A20 group (73.5% on day 220), two genera of the same family.

Lactate producers were found with high OTU abundances in the sub-communities in G16, G17, G18, G21 and G23. Specifically, *Bifidobacterium* was detected with particularly high OTU abundances during periods of high caproate concentration. *Olsenella* was present in the sub-communities G17 (17.1% day 52) and G18 (30.3% day 34) that displayed cell abundances correlating strongly positive with caprylate titres (Fig. 3). Furthermore, it showed considerably increased OTU abundances in the same sub-communities in stage 6 when the maximum caprylate concentration was detected (77.2% in G17 on day 185, 68.3% in G18 on day 202). Additionally, *Olsenella* also showed greatly increased OTU abundance during stage 6 in G23 (24.5% on day 185).

*Roseburia*, a known butyrate producer, dominated on eight of the eleven whole community analysis time points (Fig. 4) but was not present in higher abundances in most of the sorted sub-communities. *Roseburia* only showed considerable OTU abundance in sub-community G14 (30.53% on day 167, 36.83% on day 202) and was present in high OTU abundance in the predominantly negatively correlated G07 (74.43% on day 52, 18.28% on day 220). The sub-community in G27 differed in composition from all other gates and was dominated by the aerobic genus *Phyllobacterium* (93.82% on day 106, 69.31% on day 185).

The whole community sequencing and analysis of the sorted sub-communities revealed the dominance of lactate-producing organisms in the MCFA-producing microbial community and suggests links of *Bifidobacterium* with caproate production and *Olsenella* with caprylate production.

## Discussion

### The pH range determines the substrate usage and product spectrum of chain elongation

Caproate-producing microbial communities have been reported to be most effective at acidic pH around 5.5 [11]. Effective caprylate production under similar conditions was observed at pH 5.2, but was achieved by feeding liquid substrate with high ethanol to acetate ratios and in-line product extraction [34, 35]. Pure culture studies with CE model organisms have postulated growth and productivity optima at neutral pH for *Clostridium kluyveri* utilising ethanol [36] and at pH 6.05 for *Megasphaera elsdenii* utilising lactate [37]. In our community-based system, we identified pH 5.5 and 6.25 as optima for caproate and caprylate production, respectively. The pH value seems to have shaped the microbial community structure and composition as well as the community’s performance in utilising lactate and ethanol, major intermediates necessary for CE to caproate and caprylate.

Lactate was a major constituent of the substrate with concentrations as high as 74.68 g kg_TS_^−1^. In addition, lactate-producing bacteria, such as *Bifidobacterium* and *Olsenella,* were abundant in the fermentation broth and probably provided additional lactate. Despite these two rich sources, lactate was not detectable in the fermentation broth after stage 1, indicating its efficient consumption. Lactate utilisation in CE communities has been demonstrated at pH values as low as pH 5.0 [8, 38, 39]. The lactate-based chain elongation has recently been linked to the electron transferring flavoprotein (EtfAB), [40], part of a complex responsible for the likewise recently described electron bifurcation mechanism [41]. Other studies have linked lactate to increased short-chain carboxylate production [42], and an alternative lactate conversion route to propionate and acetate, the acrylate pathway, has been reported [43]. However, this pathway was not dominant in our experiment, since propionate and subsequent odd-numbered carboxylates were produced in low concentrations during most of the experiment and only increased during stages 1 and 8 (Additional file 1: S7). The constantly low lactate concentrations in the fermenter suggest that lactate-based CE is performed irrespective of the pH value. In contrast, ethanol-based CE seemed to be more effective at pH values above 5.5. This suggests that the organisms performing CE with ethanol might have a narrower preferred pH range, closer to neutral values than the lactate utilising species. Ethanol-based CE is a well described process that historically predated lactate-based CE experiments [44] in pure cultures as well as in microbial communities [11].

The consumption of reducing equivalents provided by lactate or ethanol oxidation is known to be one of the main drivers of the reverse β-oxidation [11]. With the longer-chained carboxylates resulting from reverse β-oxidation, this driving force weakens successively because the number of electrons bound with each additional step increases less (acetate binds 4 e^−^ per C, butyrate 5 e^−^ per C, caproate 5.33 e^−^ per C, caprylate 5.5 e^−^ per C, caproate 5.6 e^−^ per C; [11]). This mechanism limits the chain length of carboxylates efficiently produced by CE to eight carbon atoms.

We observed caprylate concentrations increasing nearly fivefold in stages 3 to 6 (pH 5.5 to 6.25) while the ethanol concentration concurrently decreased to below the detection limit (Fig. 1). This hints towards ethanol facilitating an additional β-oxidation step from caproate to caprylate, while caproate itself was produced utilising mainly lactate. This assumption is supported by recent studies achieving very high caprylate concentrations and specificity by feeding a substrate comprising a very high ratio of ethanol [34, 35]. Although we could not conclusively trace the metabolisation of lactate and ethanol, we suggest that lactate-based CE produced mainly caproate and was less dependent on the tested pH value range, while the utilisation of ethanol at pH values from 5.75 to 6.5 in stages 4 to 7 resulted in increased caprylate production.

The reactor pH did not only influence electron donor utilisation and chain length of the CE products but additionally affected gas production and composition, specifically methanogenesis and hydrogen production. With the 16S rRNA primers applied for amplicon sequencing, methanogenic archaea were only detected in the inoculum (*Methanobacterium* with 4.1% on day 1). After the first pH increase to 5.75 in stage 4 methanogenesis was recorded again (up to 1.1%_vol_ CH_4_), but it was constrained at low intensity. In contrast, after the pH increase to 7 in stage 8 methanogenesis was no longer inhibited and methane concentrations increased steadily (23.6% CH_4_ on day 241, Additional file 1: S4).

The hydrogen concentrations detected in our system were comparatively high (Additional file 1: S4). Hydrogen is produced by a wide range of fermentation reactions likely to simultaneously take place in the reactor, such as butyrate- and mixed acid fermentation. The observed hydrogen production reached its maximum in stage 3 (pH 5.5, 9.66 L_N_ H_2_ day^−1^ on day 99, 32.3% of the total gas production) and declined during later stages with elevated pH. This confirms published production optima for hydrogen between pH 5.5 and 6.0 [45]. In accordance, addition of lactate to biohydrogen production processes has been proven to enhance hydrogen yields [46].

### Lactic acid bacteria are the key to effective MCFA production

*Bifidobacterium* and *Olsenella* dominated in eleven of the 14 sorted sub-communities that correlated strongly positive to caproate and caprylate concentrations. Both genera produce lactate from carbohydrates, either after general amylolytic activity [47] or through the *Bifidobacterium* specific “bifid shunt” [48]. These two genera of lactic acid bacteria have been previously detected as major parts of microbial communities producing short-chain fatty acids [49–51]. They seemed to be crucial for converting maize silage polysaccharides into additional lactate, which drove the lactate-based CE and led to comparatively high MCFA concentrations in our system.

Other lactic acid producers are responsible for lactate production during the ensiling process in maize silage and are therefore highly abundant in the substrate. However, the incoming species did not prevail in our reactor, as the organisms regularly found in maize silage are *Lactobacillus* species [52, 53], which were only detected with very low abundances in our reactor (0.1%–1.2%).

In accordance with previous community-based studies [34, 35], we did not detect organisms classically linked to CE of short-chain fatty acids to MCFAs such as *Clostridium kluyveri* or *Megasphaera elsdenii*. This might be caused by abundances lower than the detection threshold or the CE being performed by other organisms generally known as carbohydrate fermenters. A major share of the short-chain carboxylates, metabolites that are elongated in the CE, were probably produced by *Roseburia,* a known butyrate-producing genus [54] highly abundant from stage 2 to 7. Despite *Roseburia* dominating the whole community, it was comparatively rarely detected in the sub-communities correlating strongly positive to caproate and caprylate concentrations. This could suggest that the butyrate provision by *Roseburia* was not a rate-limiting step in this system. Apart from CE, caproate can also be produced from galactitol, as performed by *Caproiciproducens galactitolivorans* [55]. The *Caproiciproducens* genus was consistently present in the whole community but displayed only low abundances (0.1%–1%).

The sub-community G07 correlated negatively to caproate concentration and contained *Lachnospiraceae* NK3A20 in very high abundance on day 220, when pH was 6.5 and MCFA concentrations declined while methanogenesis resumed. This may hint towards *Lachnospiraceae* NK3A20 as syntrophic MCFA-degrader under the given conditions but this assumption needs to be investigated in greater detail to allow final evaluation.

### Oxygen levels shape the chain-elongating microbial community

The daily feeding of oxic substrate led to a measureable oxygen concentration in the gas phase of the system (Additional file 1: S4). This oxygen allowed the strictly aerobic *Phyllobacterium* genus to establish and even dominate the sub-community G27. *Phyllobacterium* and other aerobic organisms probably utilised most of the influx oxygen keeping the oxygen level in the fermentation broth at a sufficiently low level for anaerobes to thrive. At the same time, the presence of oxygen might have acted as a selective pressure towards moderately oxygen-tolerant lactate producers like *Bifidobacterium* [56]. Indeed, sub-community G16, which was dominated by *Bifidobacterium* (94.77%), reached the highest overall relative cell abundance of 34.3% on day 83 with an exceptionally high oxygen concentration of 3.3%. These results suggest that the low-level oxygen influx indirectly supported MCFA production by favouring lactate formation over other more oxygen-sensitive fermentation pathways.

### Functional and structural constancy properties of the chain-elongating microbial community

The investigated microbial community remained productive, i.e. caproate and caprylate forming over a time period of 228 days from stage 2 to stage 8. It can be assumed that this functional constancy was promoted by functional redundancy of different species present in the reactor. Organisms were permanently introduced into the system together with the substrate. Furthermore, most natural microbial communities contain a large pool of rare phylotypes with abundances below 0.1%. Species from this so-called rare biosphere can potentially sustain the established community and become dominant to fill functional niches in case of disturbances [32], such as the increase in pH values. This process was accentuated by taxa emerging in stages 7 and 8 that were not prevalent before (Fig. 4). When biotechnological production systems are open and subjected to continuous in- and outflow of microorganisms, a high functional constancy of the system can be implied [57].

The investigated system was subjected to mass transfer [58] and pH value variations of varying intensity. The abrupt acidification of the neutral inoculum to pH 5.5 during reactor inoculation marked the most extreme shift of the environmental conditions and led to a considerable loss of dominant microorganisms. The pH increase to 7.0 in stage 8 led to a second major shift of the community. Both events were recorded by flow cytometry (Figs. 1, 2) as well as 16S rRNA gene amplicon sequencing (Fig. 4).

### Combining flow cytometry and amplicon sequencing for monitoring complex reactor microbiomes

Changes in community structure were detected by flow cytometric analysis and comprised both, long term trends correlating with process performance and short-term dynamics during stable performance. Amplicon sequencing delivered insights in taxonomic identity of the microorganisms and reported a remarkably unchanged core community composition during stages 2 to 7. Sequencing approaches generally provide limited information on community dynamics due to lower sampling density confined by cost, time, and workforce restrictions. Instead, flow cytometric fingerprints can closely follow community dynamics by visualising fluctuations in growth of community members and changes in abundances of distinct organisms down to the genus level [59]. The combination of both techniques via cell sorting offers a variety of benefits and can generate insight that would be impossible to conclude with each approach alone. Likely functional traits of certain sub-communities can be concluded by correlation analysis and then verified by sequencing. In the exemplary case of G16, the correlation analysis suggested a role beneficial for caproate production that could be assigned to *Bifidobacterium*. Finally, flow cytometric cell sorting enables a higher resolution of sequencing analysis with a given number of reads, because it reduces the species richness in sub-communities of interest.

### MCFA production in a sustainable bio-economy

Microbial MCFA production is very likely part of future bio-refineries, the keystones of the circular bio-economy. Such bio-refineries will utilise complex solid biomass, as well as organic waste streams, and produce a range of valuable products while interlinking material and energy streams from different processes to maximise efficiency and profitability.

We could show that the utilisation of complex solid biomass for MCFA production did not necessitate any costly pre-treatments, such as saccharification. The separate ethanol addition implemented in most experiments was identified as a major hurdle to economic feasibility in large scale applications [17]. Reportedly, the external production and provision of ethanol accounted for at least 20% of the life cycle impacts of MCFA production. The authors suggested stimulating in situ ethanol formation and substituting ethanol with alternative carbon sources and electron donors. In this study, we focused on a lactic acid rich substrate additionally containing ethanol to overcome this issue. To further enhance MCFA production, this substrate was added with high organic loading rates (up to 21.01 g_VS_ L^−1^ day^−1^), which led to comparatively low degrees of substrate degradation (averaged 26.9% ± 8.5%, Additional file 1: S3). The degrees of substrate degradation could potentially be increased by further optimising process parameters, such as the HRT. Additionally, still non-degraded substrate could be utilised for biogas production in a subsequent anaerobic digestion after the carboxylate separation. The separation procedure will likely produce a mixture of different chain length fatty acids. After fractionating the stream, short-chain fatty acids could be recirculated to serve as substrates for CE. However, they could also be spiked into the methanogenic digester for fast biogas production and thereby demand-driven electricity production. Depending on the supply situation in the grid and current MCFA prices, such a system could switch between operational modes to maximise profitability.

### Outlook

Future reactor designs including inline product recovery might greatly enhance product yields by reducing product inhibition and shifting reaction equilibria in favour of MCFAs. Because of its low solubility [11] and the comparatively high concentrations achieved with the presented reaction system, caprylate may be particularly promising with regard to product separation. Electrochemical approaches promise to be beneficial for this task, as they might enable acidification for phase separation of the undissociated acids on one electrode and supplement the base addition for pH control on the other [60].

Such an approach would rely on fast, heuristic process control mechanisms to steer the community-based process. Such mechanism could be provided by a robust sample treatment protocol, on-line flow cytometry [29] and automated data processing (CHIC [61]; flowCyBar [33]) combined to form a “community sensor” with faster response times than provided by traditional abiotic process parameters. This community sensor would utilize the state, recent history and dynamic trends of the microbial community structure to determine the necessity of changing process parameters, such as the pH and the oxygen concentration. Conceivable scenarios include a temporary decrease of the pH value to counter a proliferating methanogenic subcommunity or the controlled spiking of small amounts of air to establish a selective pressure in favour of moderately oxygen-tolerant lactate producers.

## Conclusion

The production of MCFAs from complex, lactate-containing substrate without the addition of external electron donors is possible with high titres and yields. Caproate production was optimal at pH 5.5 and linked to lactate-based chain elongation, while the highest caprylate concentration was reached at pH 6.25 and connected to ethanol-based chain elongation. Lactate-producing organisms played a major role in supporting the MCFA production. The process was realised in a well-established reactor design, which will greatly ease scaling and profitable implementation of the process. Flow cytometric single cell analysis enabled fast analysis and evaluation of structure, function and dynamics of the MCFA-producing microbial community.

## Methods

### Fermenter configuration and operational parameters

A 15 L continuous stirred-tank reactor (Bräutigam Kunststoffsysteme GmbH, Mohlsdorf, Germany, Additional file 1: S1) equipped with an S-shape overhead stirrer (75 rpm, RZR2102, Heidolph Instruments, Schwabach, Germany) was operated with 12 L working volume at 38 °C (MA–4 Umwälzthermostat, Julabo, Seelbach, Germany).

The reactor was inoculated with 6 L acidogenic percolate from a previous study [39] and 6 L methanogenic digestate from a pilot-scale biogas plant (DBFZ—Deutsches Biomasseforschungszentrum gGmbH, Leipzig, Germany) performing mono-digestion of maize silage. The inocula were mixed under aerobic conditions and adjusted to pH of 5.5 by adding 120 mL of 10 M hydrochloric acid.

The reactor was fed with 3 L substrate every 24 h resulting in a stable hydraulic retention time (HRT) of 4 days. The feed consisted of maize silage (from a farm in Neichen, Germany), which was mixed with pre-warmed deionised water and supplemented with 1 g L^−1^ trace element solution and 4.5 g L^−1^ ammonium bicarbonate solution (Carl Roth, Karlsruhe, Germany) (Additional file 1: S2). The organic loading rate ranged from 14.4 to 21.0 g_VS_ L^−1^ day^−1^ (Additional file 1: S5). Two 5 L gasbags were connected to the headspace to compensate pressure fluctuations due to filling level variations during the feeding process. The pH value was controlled using a sensor (pH 3110, WTW, Weilheim, Germany), a control unit (InPro 325X (ISM), Mettler Toledo, Columbus, USA) and a peristaltic pump (Pumpdrive 5201, Heidolph Instruments) dispensing 10 M sodium hydroxide solution (Sigma Aldrich, Steinheim, Germany).

### Analysis of abiotic parameters

The total gas production was measured and displayed continuously by a drum type gas flowmeter (TG05/5, Ritter Apparatebau, Bochum, Germany). It was corrected to standard conditions (273.15 K, 1.01325 bar) according to [10]. The gas composition was determined as previously described by [14]. Total solids and volatile solids (VS) of fresh fermentation broth and maize silage were measured according to standard procedures [62–64] and corrected according to [65]. Total ammonia nitrogen (TAN) concentration (Nessler’s method according to [66], DR3900, Hach Lange, Loveland, USA), the concentrations of organic acids and alcohols [14], Additional file 1: S6, head space GC System 7890 A, Agilent Technologies, Santa Clara, USA) and the electrical conductivity (Cond3310, WTW) of the supernatant of fermentation broth and maize silage eluate were analysed. For this, 25 g maize silage was eluted with 250 mL deionised water for 24 h and fermentation broth and maize silage eluate were centrifuged at 10,000×*g* for 10 min at 10 °C (Megafuge, Hanau, Germany).

### Sample fixation for single cell analysis, sorting and sequencing

Fermentation broth samples (200 µL) were taken with a clipped 1-mL pipette tip and handled according to [67]. In short, the samples were transferred to 2-mL tubes (Eppendorf AG, Hamburg, Germany), suspended in 1.5 mL phosphate buffered saline (PBS, 1.8 g L ^1^ Na_2_HPO_4_, 0.223 g L^−1^ NaH_2_PO_4_, 8.5 g L^−1^ NaCl in deionised H_2_O pH 7.2) and sonicated for 1 min (Banlin electronic, Berlin, Germany) to break up cell clusters and detach cells from substrate constituents. The samples were filtered using 50-µm CellTrics (Sysmex Partec GmbH, Görlitz, Germany) and separated into three 400 µL aliquots. The aliquots were centrifuged twice for 5 min at 4000×*g* and 10 °C. After removing the supernatant completely, the formed pellets were subsequently dried for 40 min under infrared radiation at 2500×*g*, 35 °C, and − 97 kPa vacuum (IR MICRO-CENVAC, N-Biotec, Bucheon-si, South Korea) and stored at room temperature in the dark until flow cytometric measurement and/or cell sorting. Samples were stable for at least 6 months. All solutions used for cell treatment were cleaned of any particles using 0.2-µm syringe filters (Eppendorf AG).

### Staining of fixed cells

For cell staining the samples were handled according to [67]. In short, the resuspended cells were washed in 1.5 mL PBS at 4000×*g* for 5 min, sonicated for 1 min and filtered using 50-µm CellTrics. DAPI staining was applied according to [33]. The samples were diluted to an optical density of 0.035 at 700 nm (d_cuvette_ = 0.5 cm), incubated 20 min in 1 mL 4.1 mmol L^−1^ Tween 20 and 0.11 mol L^−1^ citric acid and afterwards stained in 2 mL 0.24 µmol L^−1^ DAPI (Sigma Aldrich, St. Louis, USA) solution for 24 h in the dark.

### Flow cytometric measurement and data evaluation

The cytometric measurements were performed using a MoFlo Legacy cell sorter (Beckman-Coulter, Brea, USA) equipped with a blue 488 nm Genesis MX488-500 STM OPSL (400 mW, Coherent, Santa Clara, USA) and a 355 nm UV Xcyte CY-SM150 laser (150 mW, Lumentum, Milpitas, USA). The blue laser induced the FSC (bandpass filter 488 nm ± 5 nm, neutral density filter 1.9) related to cell size, and the side scatter (SSC, same filter set) related to cell density and used as the trigger signal. The 355 nm laser excited the DAPI fluorescence (bandpass filter 450 nm ± 32.5 nm) related to the cellular DNA content. Hamamatsu photomultiplier tubes (Models R928 and R3896; Hamamatsu Photonics, Hamamatsu City, Japan) were used for scatter and fluorescence light detection. The fluidic system was run at 56 psi (3.86 bar) with sample overpressure at maximum 0.3 psi and a 70 μm nozzle. The sheath fluid consisted of 10× sheath buffer (19 mmol L^−1^ KH_2_PO_4_, 38 mmol L^−1^ KCl, 166 mmol L^−1^ Na_2_HPO_4_, 1.39 mol L^−1^ NaCl in deionised H_2_O) diluted to a 0.2 × working solution (for cell sorting: 0.5 × working solution) with 0.1 μm filtered deionised H_2_O. The cytometer was operated using Summit 4.3 (Beckman-Coulter). Calibration of the cytometric set-up in the linear range was performed using 1 µm blue fluorescent (F–8815) and 2 µm yellow-green fluorescent FluoSpheres (F8827, Molecular Probes, ThermoFisher Scientific, Waltham, USA). The instrument was calibrated in the log range using blue fluorescent beads (0.5 μm and 1 μm, Fluoresbrite BB Carboxylate microspheres, 360 nm excitation-maximum, 407 nm emission maximum, PolyScience, Niles, USA), which were also added to each sample to ensure the measurement stability. *Escherichia* *coli* BL21 (DE3) was used as biological standard for control of every staining procedure (dot plots of beads and *E.* *coli* in Additional file 1: S9). The stained samples were filtered using 50-μm CellTrics and analysed at an acquisition rate of 3000 events per second until 250,000 events were recorded in the cell gate (gating strategy in Additional file 1: S8). During cell sorting, 500,000 cells per sub-community were acquired using the most accurate “single and one-drop” mode (purity 99%) at acquisition rates not higher than 2500 events per second. The cells were collected in a pellet (centrifugation at 20,000×*g*, 6 °C for 25 min, supernatant removed) and stored at − 20 °C for a maximum of 9 months.

The cytometric data were evaluated using FlowJo v10.0.8r1 with the engine v3.04910 (FlowJo, LLC, Ashland, USA) and the R packages flowCHIC [33] (https://bioconductor.org/packages/release/bioc/html/flowCHIC.html) and flowCyBar [31] (https://bioconductor.org/packages/release/bioc/html/flowCyBar.html). NMDS plots based on the Bray–Curtis dissimilarity of the samples’ flow cytometric fingerprints were used to illustrate the microbial community dynamics. The frequency distribution of the relative cell abundances of 31 sub-communities (Additional file 1: S8) was visualised in boxplots, while their temporal development was recorded in maps with 21 shades colour-coding deviation from their mean value. Correlation matrices based on Spearman’s rank order coefficient and corrected significance [68] were used to identify sub-communities of interest for cell sorting and 16S rRNA amplicon sequencing for taxonomic analysis (Additional file 1: S12).

### 16S rRNA gene amplicon sequencing

The whole microbial community was examined at eleven time points from fixed, non-sorted samples by amplicon sequencing of 16S rRNA genes (Additional file 1: S13). Furthermore, eight sub-communities were sorted from two time points each (Additional file 1: S12), i.e. from the days 24 (G07), 34 (G18), 52 (G16, G17, G23), 83 (G16), 106 (G21, G27), 141 (G21), 167 (G14), 185 (G17, G23, G27), 202 (G14, G18), 220 (G07) and the sorted cells were subsequently used for sequencing. DNA extraction was performed according to [69] using 70 µL of 10% Chelex 100 solution (Biorad, Hercules, USA) for 70 µL of whole community sample (OD 0.01, d = 5 mm, ʎ = 700 nm) and 500,000 cells of the sorted sample. The extracted DNA was stored at − 20 °C. The library was created according to [69]. In short, the V3 and V4 regions of the 16S rRNA genes were amplified with Pro341F 5′-CCTACGGGNBGCASCAG-3′ [70] and Pro805R 5′-GACTACNVGGGTATCTAATCC-3′ [71] primers in an 18 and 20 cycle PCR for sorted and whole community samples, respectively. Subsequently, ten cycles of a second PCR were performed with barcoded primers. Paired-end sequencing was performed on an Illumina MiSeq sequencer with the v3 kit, 2 × 300 bp, 600 cycles (Illumina, San Diego, USA) by DSMZ (Braunschweig, Germany). The dataset was processed and evaluated using Mothur 1.39 [72] and UCHIME [73]. An abundance threshold of 0.1% was set for defining OTUs. SILVA v128 was used for taxonomic affiliation. PCR, sequencing and data analysis pipeline were controlled by including a mock community [74]. Details concerning DNA extraction, PCR, sequencing, the data processing steps and the mock community are explained in Additional file 1: S13.

## Additional files

**Additional file 1.** Additional information containing details about: **S1** the reactor set-up, **S2** the feed composition, **S3** the degree of substrate degradation, **S4** the gas production and composition, **S5** miscellaneous reactor parameters, **S6** the gas chromatography detection and calibration limits, **S7** the concentrations of non-target carboxylates, **S8** the gating strategy, **S9** the flow cytometric controls, **S10** the microbial community dynamics, **S11** the correlation analysis, **S12** the flow cytometric cell sorting and **S13** the sequencing protocols and details of sequence analysis.

**Additional file 2.** Additional information containing a short movie in a.gif file with the cytometric fingerprints of all 89 time points as 500 ms frames.

## Data Availability

The flow cytometry dataset generated and analysed during the current study is available in the FlowRepository under the ID: FR-FCM-ZYL3. The DNA sequences generated and analysed in the study are available on NCBI under the Bioproject Number PRJNA504543.

## Abbreviations

CE: chain elongation
DAPI: 4′,6-diamidino-2-phenylindole
FSC: forward scatter
HRT: hydraulic retention time
MCFA: medium-chain fatty acid
NMDS: non-metric multidimensional scaling
OTU: operational taxonomic unit
PBS: phosphate buffered saline
VS: volatile solids
