# Supplementary material for: Key sub-community dynamics of medium-chain carboxylate production
Source: Microb Cell Fact. 2019 May 28;18:92. doi: 10.1186/s12934-019-1143-8 (PMC6537167; doi:10.1186/s12934-019-1143-8)
Supplement: Supplementary file 1 — Additional file 1. Additional information containing details about: S1 the reactor set-up, S2 the feed composition, S3 the degree of substrate degradation, S4 the gas production and composition, S5 miscellaneous reactor parameters, S6 the gas chromatography detection and calibration limits, S7 the concentrations of non-target carboxylates, S8 the gating strategy, S9 the flow cytometric controls, S10 the microbial community dynamics, S11 the correlation analysis, S12 the flow cytometric cell sorting and S13 the sequencing protocols and details of sequence analysis. [file 12934_2019_1143_MOESM1_ESM.pdf]

Additional file 1 for

Key sub-community dynamics of medium-chain carboxylate production

Johannes Lambrecht, Nicolas Cichocki, Florian Schattenberg, Hauke Harms, Susann Müller and Heike Sträuber
Department of Environmental Microbiology, Helmholtz Centre for Environmental Research, Permoserstr. 15, 04318 Leipzig, Germany

Content:

S1:	Fermenter system setup	2
S2:	Feed composition	3
S3:	Substrate degradation	6
S4:	Gas production and composition	6
S5:	Miscellaneous reactor parameters	8
S6:	Gas chromatography detection and calibration limits	9
S7:	Concentrations of non-target carboxylates	10
S8:	Flow cytometric analysis - gating strategy	11
S9:	Flow cytometric analysis - controls	12
S10:	Flow cytometric analysis - microbial community dynamics	13
S11:	Correlation analysis	18
S12:	Flow cytometric cell sorting	16
S13:	Sequencing protocols and details	13
References		33

S1: Fermenters system setup

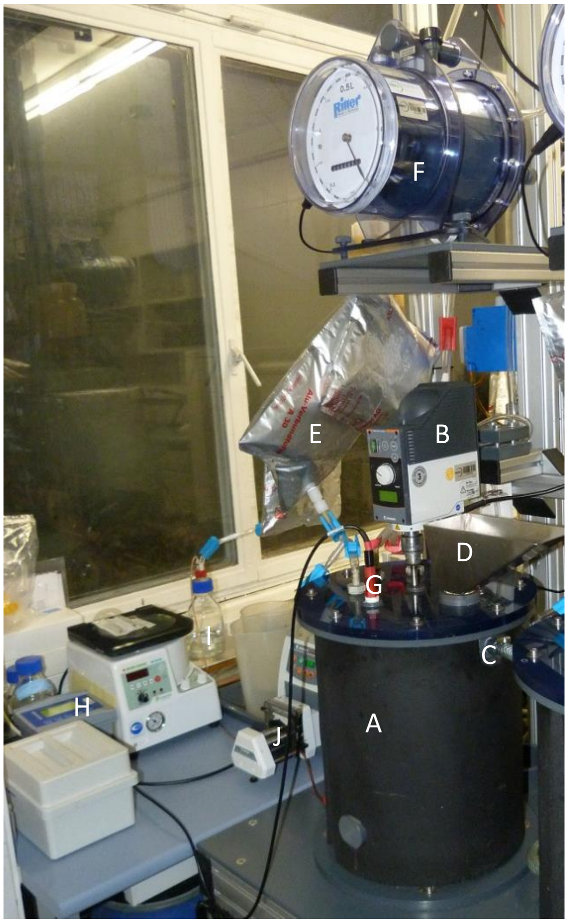

Figure S1: Reactor system consisting of **A** fermenter vessel, **B** overhead stirrer, **C** heating system, **D** substrate input port, **E** gasbag, **F** gas flow meter, **G** pH-sensor, **H** pH controller, **I** sodium hydroxide reservoir, **J** peristaltic pump and waste port below the fermenter vessel. The fermenter was run semicontinuously and fed every 24 h. Prior to the feeding procedure 4 L of the fermentation broth was drained and recirculated into the feeding port to prevent build up of a floating layer and to homogenise the fermentation broth.

S2: Feed composition

Corn Silage

Over the course of the experiment three different corn silage batches from a nearby farm in Neichen, Germany, were fed into the fermenter. The first batch was fed from day 1 to 55, the second from day 56 to 139, and the third from day 140 to the end on day 241. The freshly obtained corn silage was manually compressed in 80 L plastic barrels with gastight caps and stored at 4 °C. The barrels were opened biweekly to prepare the daily substrate portions and the barrel headspace was flushed with nitrogen to reduce the oxygen exposure and the risk of mould fungus infestation. The prepared substrate was stored in vacuum bags at 4 °C. The soluble (Table 1 S2) and insoluble (Table 2 S2) compounds of the substrate as well as its pH value were analysed.

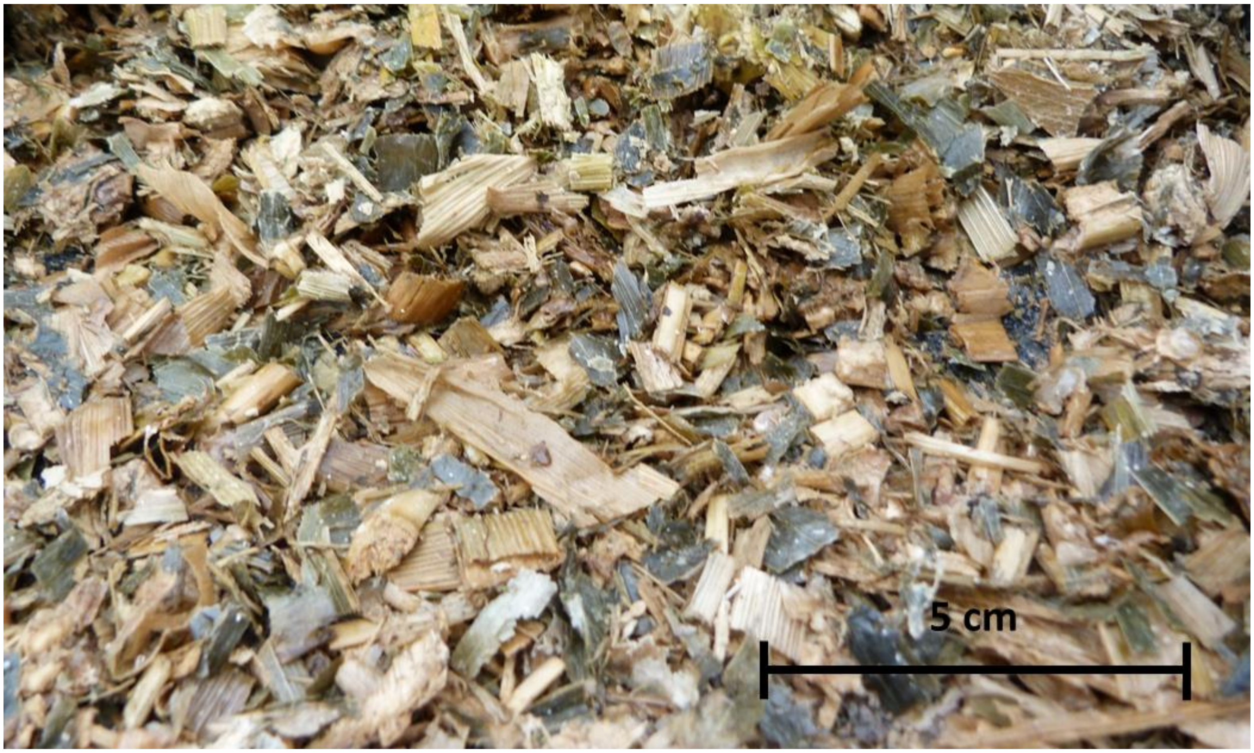

Figure S2: Exemplary picture of the corn silage fed to the fermenter.

Table 1 S2: Composition of soluable substrate components of the three corn silage batches fed into the reactor over the course of the experiment. The analytes were determined in eluate of the substrate (25 g wet substrate was eluated in 250 mL distilled water for 24 h at 22 °C [1]) and measured by headspace gas chromatography according to pubslished protocols [2], detection and calibration limits in Additonal file 1 S6). The measurements were perfomed in triplicates.

Component	Substrate batch 1 (days 1 to 56)		Substrate batch 2 (days 57 to 140)		Substrate batch 3 (days 141 to 241)	
	($g\ kg_{TS}^{-1}$)	(± standard dev.)	($g\ kg_{TS}^{-1}$)	(± standard dev.)	($g\ kg_{TS}^{-1}$)	(± standard dev.)
Formate	0.14	(± 0.01)	0.12	(± 0.00)	0.12	(± 0.00)
Acetate	61.87	(± 1.54)	31.91	(± 0.07)	14.02	(± 0.08)
Propionate	7.26	(± 0.22)	3.18	(± 0.06)	0.00	(± 0.00)
i-Butyrate	0.03	(± 0.00)	0.05	(± 0.00)	0.09	(± 0.00)
n-Butyrate	0.49	(± 0.01)	1.95	(± 0.01)	0.02	(± 0.00)
i-Valerate	0.04	(± 0.00)	0.05	(± 0.00)	0.04	(± 0.00)
n-Valerate	0.04	(± 0.00)	0.24	(± 0.00)	0.00	(± 0.00)
n-Caproate	0.08	(± 0.00)	0.14	(± 0.00)	0.02	(± 0.00)
n-Caprylate	0.00	(± 0.00)	0.01	(± 0.00)	0.00	(± 0.00)
Lactate	59.77	(± 2.79)	74.68	(± 0.95)	66.89	(± 1.92)
2-Propanol	0.12	(± 0.00)	0.02	(± 0.00)	0.00	(± 0.00)
Ethanol	14.09	(± 0.29)	15.35	(± 0.06)	9.49	(± 0.07)
2-Butanol	0.21	(± 0.00)	0.09	(± 0.00)	0.02	(± 0.00)
1-Propanol	11.76	(± 0.27)	2.21	(± 0.01)	0.03	(± 0.00)
1-Butanol	0.03	(± 0.00)	0.15	(± 0.00)	0.00	(± 0.00)

Table 2 S2: Composition of the corn silage batches fed into the reactor over the course of the experiment as determined by substrate analysis according to standard procedures [3,4]. The pH value, total solids (TS), volatile solids (VS) corrected according to [5], raw water, raw ash, raw protein, raw fat, raw fiber, nitrogen free extractives (NfE), non-fibrous carbohydrates (NFC), hemicellulose, cellulose, lignin, neutral detergent fiber (NDF) and acid detergent fiber (ADF) are given.

						Batch 1	Batch 2	Batch 3	
					**pH**		3.75	3.86	
					**TS**	30.74	25.71	36.19	%
					**VS**	94.02	96.13	97.82	$\%_{TS}$
					**Raw water**	69.44	75.29	64.35	%
					**Raw ash**	60.16	101.67	32.12	$g\ kg_{TS}^{-1}$
					**Raw protein**	95.08	79.47	82.99	$g\ kg_{TS}^{-1}$
					**Raw fat**	11.01	8.91	13.21	$g\ kg_{TS}^{-1}$
					**Raw fiber**	220.57	264.90	197.35	$g\ kg_{TS}^{-1}$
TS	VS	carbohydrates			**NfE**	613.18	545.06	674.33	$g\ kg_{TS}^{-1}$
					**NFC**	167.49	173.04	438.81	$g\ kg_{TS}^{-1}$
					**Hemicellulose**	315.06	370.25	223.40	$g\ kg_{TS}^{-1}$
			NDF		**Cellulose**	284.37	215.86	167.73	$g\ kg_{TS}^{-1}$
				ADF	**Lignin**	66.82	50.81	41.74	$g\ kg_{TS}^{-1}$
					**NDF**	666.26	636.92	432.87	$g\ kg_{TS}^{-1}$
					**ADF**	351.20	266.67	209.47	$g\ kg_{TS}^{-1}$

Trace elements

1 mL L^{-1} trace element solution was added to the feed. The trace element solution consisted of 1.5 g L^{-1} $FeCl_2 \times 4\ H_2O$, 6 mg L^{-1} H_3BO_3, 190 mg L^{-1} $CoCl_2 \times 6\ H_2O$, 100 mg L^{-1} $MnCl_2 \times 4\ H_2O$, 70 mg L^{-1} $ZnCl_2$, 36 mg L^{-1} $Na_2MoO_4 \times 2\ H_2O$, 24 mg L^{-1} $NiCl_2 \times 6\ H_2O$, 2 mg L^{-1} $CuCl_2 \times 2\ H_2O$ and 3 mg L^{-1} Na_2SeO_3 x H_2O.

Water

Depending on the TS and VS content of the respective corn silage batch, the daily feed was supplemented with 2.08 L to 2.35 L distilled H_2O pre-warmed to 38 °C.

Ammonium source

4.5 g L^{-1} d^{-1} ammonium bicarbonate (Carl Roth, Karlsruhe, Germany) was added to the feed from day 35 on. At this point the inoculum's total ammonium nitrogen (TAN) load (0.77 g L^{-1}) was depleted (0.06 g L^{-1}) and urea (up to 10 g L^{-1} d^{-1}) added before did not result in increased TAN concentrations.

S3: Substrate degradation

Table S3: Average degrees of substrate degradation during the experimental stages with their respective standard deviation. VS values in the fermentation broth were measured two to three times a week. The degrees of degradation was calculated according to [6] and are shown as averages over an experimental stage.

**Experimental stage**	**Average VS degradation** (± standard dev.)	
2 TAN-shortage	20.9%	(± 7.2)
3 Consolidation	25.6%	(± 7.5)
4 ph 5.75	22.5%	(± 8.7)
5 pH 6.0	31.9%	(± 13.5)
6 pH 6.25	27.9%	(± 8.4)
7 pH 6.5	27.5%	(± 7.9)
8 pH 7.0	32.0%	(± 6.5)
Mean	26.9%	(± 8.5)

S4: Gas production and composition

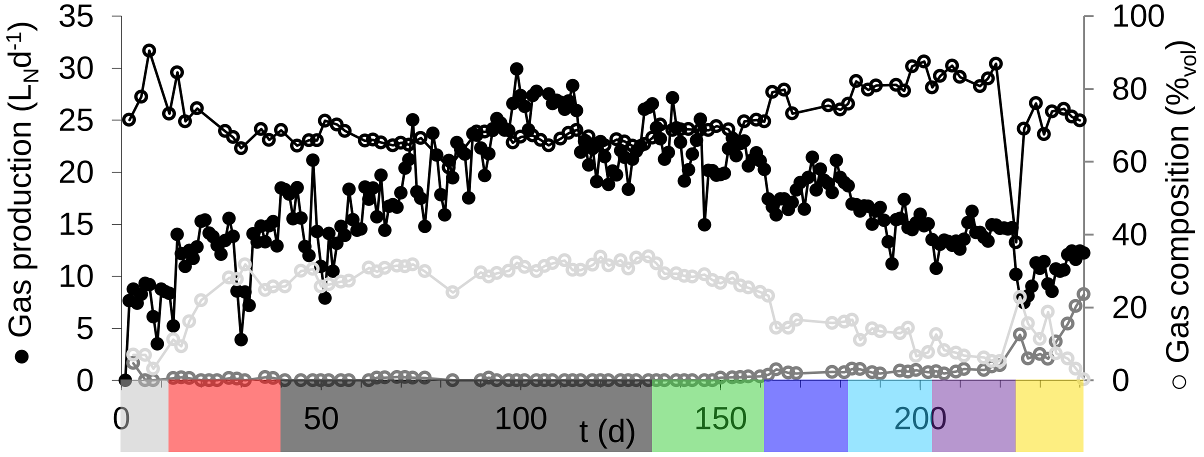

Figure 1 S4: Gas production as standard volume ● and gas composition over the course of the experiment. H_2 ○, CH_4 ○ and CO_2 ○ are shown. The respective percentages missing to 100% are constituted by N_2 and O_2. The time periods of the eight experimental stages are colour-coded along the x axis: 1 - start-up ●, 2 - TAN-shortage ●, 3 - consolidation ●, 4 - pH 5.75 ●, 5 - pH 6.0 ●, 6 - pH 6.25 ●, 7 - pH 6.5 ● and 8 - pH 7 ●.

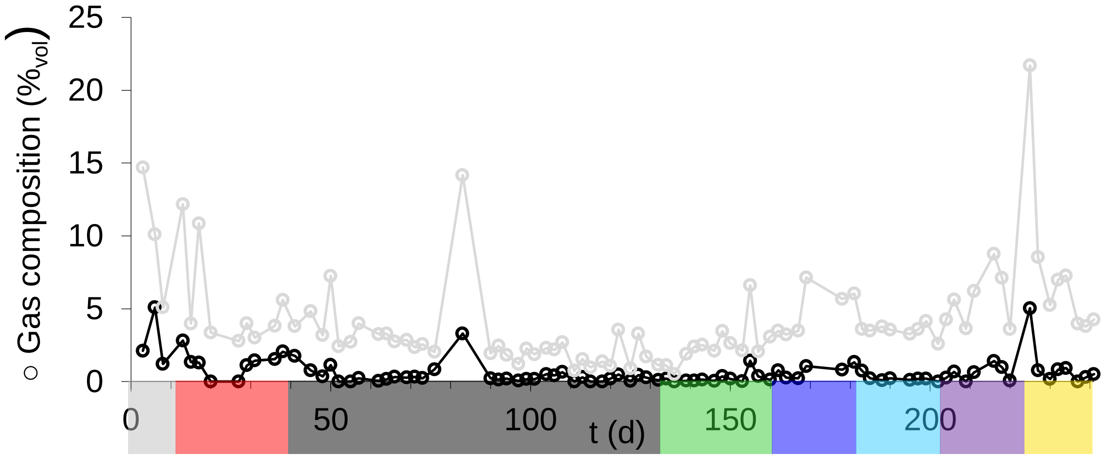

Figure 2 S4: Proportions of N_2 ○ and O_2 **O** in the gas phase over the course of the experiment. The time periods of the eight experimental stages are colour-coded along the x axis: 1 - start-up ●, 2 - TAN-shortage ●, 3 - consolidation ●, 4 - pH 5.75 ●, 5 - pH 6.0 ●, 6 - pH 6.25 ●, 7 - pH 6.5 ● and 8 - pH 7 ●.

S5: Miscellaneous reactor parameters

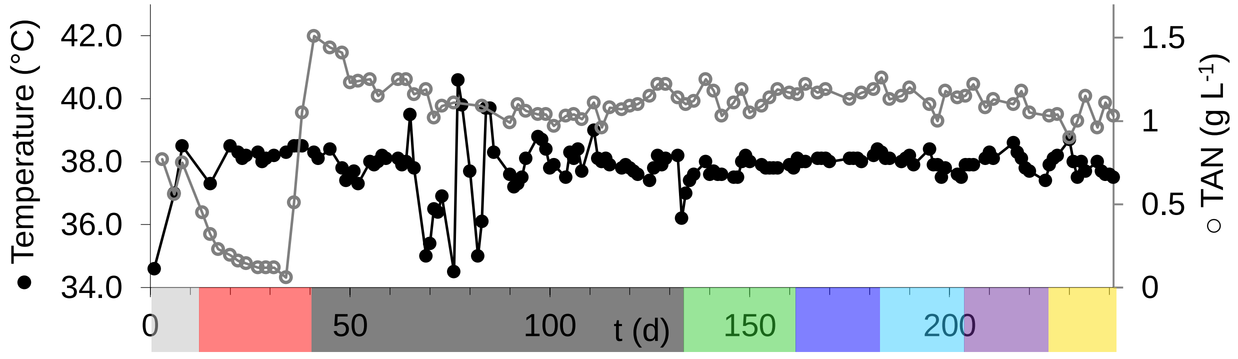

Figure 1 S5: Temperatue ● and Total ammonum nitrogen (TAN) ○. The time periods of the eight experimental stages are colour-coded along the x axis: 1 - start-up ●, 2 - TAN-shortage ●, 3 - consolidation ●, 4 - pH 5.75 ●, 5 - pH 6.0 ●, 6 - pH 6.25 ●, 7 - pH 6.5 ● and 8 - pH 7 ●.

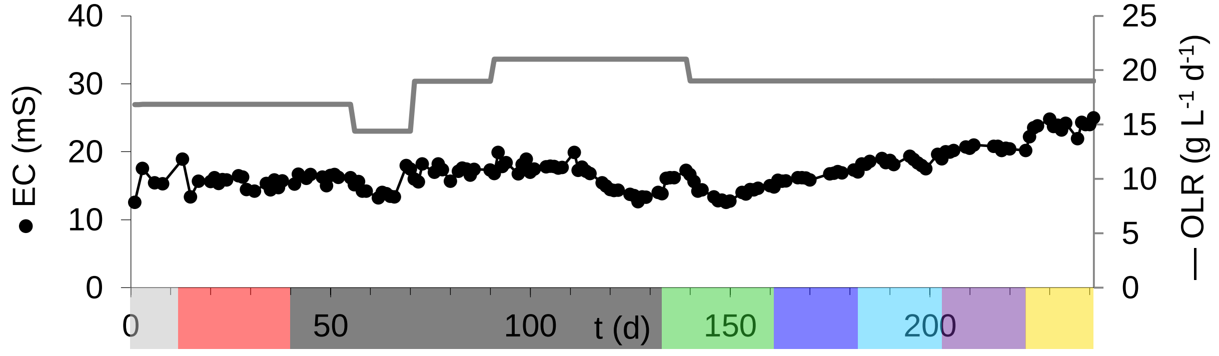

Figure 2 S5: Electric conductivity ● and organic loading rate (OLR) —. The time periods of the eight experimental stages are colour-coded along the x axis: 1 - start-up ●, 2 - TAN-shortage ●, 3 - consolidation ●, 4 - pH 5.75 ●, 5 - pH 6.0 ●, 6 - pH 6.25 ●, 7 - pH 6.5 ● and 8 - pH 7 ●.

S6: Gas chromatography detection and calibration limits

Table S6: Monitored analytes with their respectable total detection limit, i.e. the lowest concentration detectable, as well as lower and upper calibration limits, which mark the range of reliable measurements. Analyses were performed according to previously published protocols [2]. The compounds of interest and major interest are shaded.

**Compound**		(mg L^{-1})		
		Detection limit	Calibration limits	
			lower	upper
Formic acid	Formate	1.48	4.92	1220
Acetic acid	Acetate	0.90	2.99	1044.6
Propionic acid	Propionate	6.13	20.42	990
i-Butyric acid	i-Butyrate	0.03	0.10	948
n-Butyric acid	n-Butyrate	0.03	0.10	960
i-Valeric acid	i-Valerate	0.01	0.06	930
n-Valeric acid	n-Valerate	0.028	0.095	930
i-Caproic acid	i-Caproate	0.039	11.651	1165
n-Caproic acid	n-Caproate	0.019	0.063	465
Heptanoic acid	Heptanoate	0.019	13.317	1331
Caprylic acid	Caprylate	0.02	0.16	180
Nonanoic acid	Nonanoate	0.01	0.07	80
Decanoic acid	Decanoate	0.01	0.02	20
Lactic acid	Lactate	13.32	44.4	1051
Benzoic acid	Benzoate	0.603	2.01	1004.8
Phenylacetic acid	Phenyl acetate	1.199	3.996	999.4
Phenylpropionic acid	Phenyl propionate	1.652	5.507	1001.2
Ethanol		0.074	0.395	1185
1-Propanol		0.436	1.455	1200
2-Propanol		0.435	1.451	1170
1-Butanol		0.083	0.405	1215
2-Butanol		0.095	0.405	1215

S7: Concentrations of non-target carboxylates

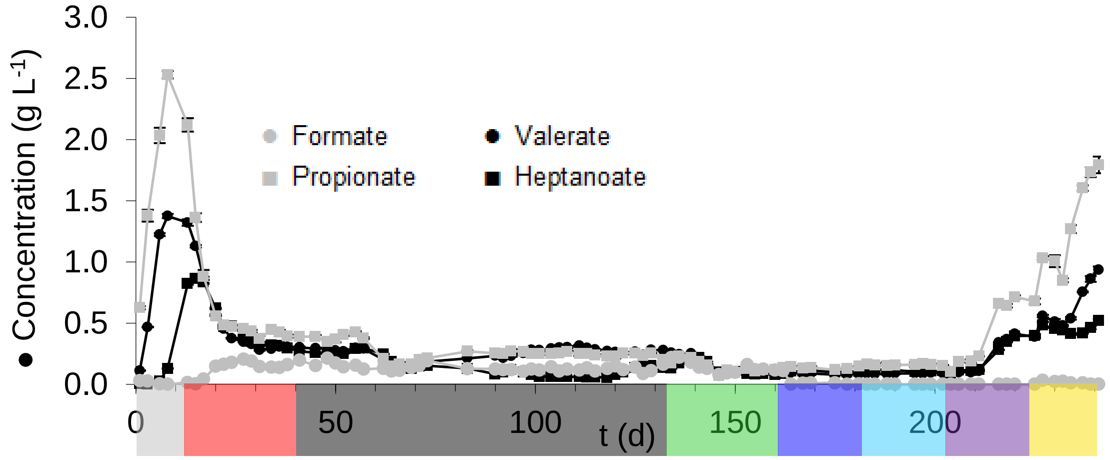

Figure 1 S7: Concentrations of the odd-numbered carboxylates formate ●, propionate ■, valerate ● and heptanoate ■ over the course of the experiment. The time periods of the eight experimental stages are colour-coded along the x axis: 1 - start-up ●, 2 - TAN-shortage ●, 3 - consolidation ●, 4 - pH 5.75 ●, 5 - pH 6.0 ●, 6 - pH 6.25 ●, 7 - pH 6.5 ● and 8 - pH 7 ●.

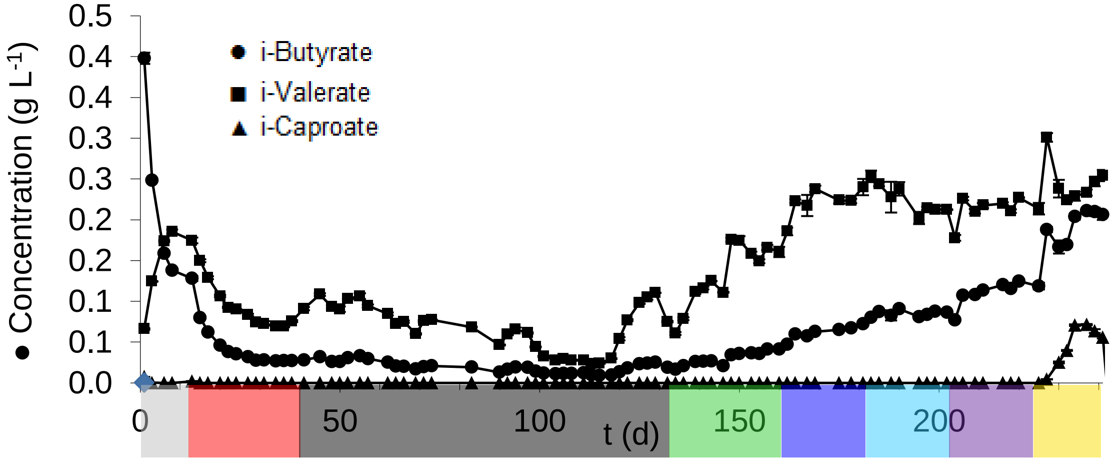

Figure 2 S7: Concentrations of the iso-carboxylates i-butyrate ●, i-valerate ■ and i-caproate ▲ over the course of the 241 day experiment. The time periods of the eight experimental stages are colour-coded along the x axis: 1 - start-up ●, 2 - TAN-shortage ●, 3 - consolidation ●, 4 - pH 5.75 ●, 5 - pH 6.0 ●, 6 - pH 6.25 ●, 7 - pH 6.5 ● and 8 - pH 7 ●.

S8: Flow cytometric analysis - gating strategy

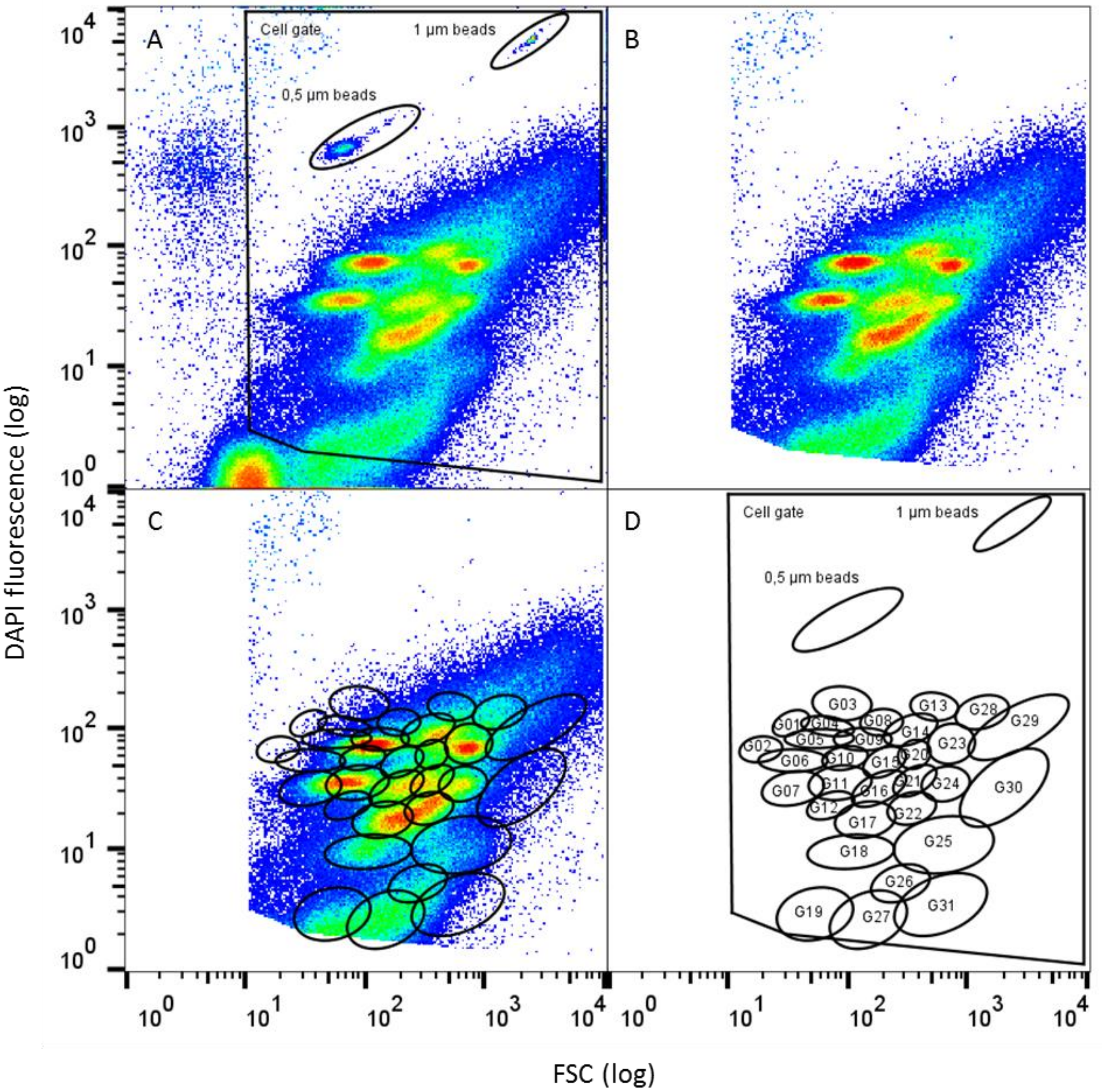

Figure S8: Master gate template composed of 1) the bead gates, 2) the cell gate that includes exactly 250,000 cells (excluding the bead gates) and 3) the gates G01 to G31 marking the respective sub-communities. **A** shows the complete fingerprint of day 164 as it is measured with the cell gate and the bead gates. **B** shows the 250,000 cells in the cell gate used for further analysis. **C** shows the cells used for further analysis with the sub-community gates G01 to G31. The sub-community gates have been generated considering all 89 samples. **D** shows the complete master gate template with named gates.

S9: Flow cytometric analysis - controls

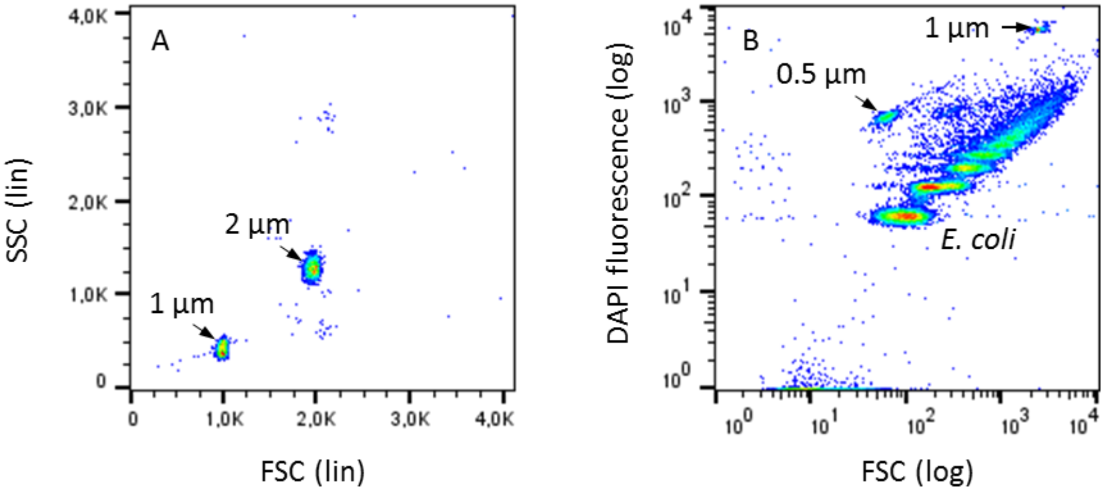

Figure S9: Beads used for daily calibration and control of measurement stability and biological standard used as a staining control. **A** shows the 1 µm blue fluorescent (F-8815) and 2 µm yellow-green fluorescent FluoSpheres (F8827, Molecular Probes, ThermoFisher Scientific, Waltham, Massachusetts, USA) used for initial instrument calibration in the linear scale. **B** shows blue fluorescent 0.5 µm and 1 µm Fluoresbrite BB Carboxylate microspheres (360/407, PolyScience, Niles, Illinois, USA), which were used for subsequent calibration in the log range and later added to every measured sample and *E. coli* BL21 (DE3) stained with every batch of samples to control the staining procedure.

S10: Flow cytometric analysis - microbial community dynamics

Table S10: Relative cell abundances of the 31 sub-communites of the master gate template (gating strategy in Additonal file 1 S8) over all sampling points in %. The time periods of the eight experimental stages are colour-coded along the x axis: 1 - start-up ●, 2 - TAN-shortage ●, 3 - consolidation ●, 4 - pH 5.75 ●, 5 - pH 6.0 ●, 6 - pH 6.25 ●, 7 - pH 6.5 ● and 8 - pH 7 ●. The relative cell abundances of sorted sub-communities are shaded in light green (Additonal file 1 S 11, 12).

	Mean	SD	Day: 1	3	6	8	13	15	17	20	22	24	27	29	31	34	36	38
**G01**	0.09	0.22	0.21	0.12	0.02	0.02	0.00	0.02	0.01	0.01	0.02	0.02	0.03	0.01	0.01	0.00	0.01	0.01
**G02**	0.15	0.49	0.35	0.21	0.04	0.03	0.00	0.01	0.01	0.01	0.03	0.02	0.03	0.02	0.03	0.01	0.01	0.01
**G03**	0.21	0.25	0.18	0.2	0.14	0.09	0.02	0.04	0.03	0.03	0.03	0.03	0.09	0.01	0.02	0.02	0.02	0.02
**G04**	0.34	0.42	0.62	0.53	0.2	0.25	0.04	0.09	0.04	0.09	0.07	0.07	0.28	0.04	0.04	0.04	0.07	0.05
**G05**	1.95	1.84	0.89	1.34	0.49	1.57	0.12	0.42	0.63	1.07	0.88	1.1	1.62	0.31	0.23	0.33	0.34	0.3
**G06**	1.57	2.69	2.26	2.09	0.68	1.23	0.17	0.27	0.46	0.57	1.18	1.16	1.12	0.94	1.03	0.62	0.64	0.62
**G07**	6.07	4.98	3.16	5.55	6.71	10.8	1.58	3.19	7.06	7.47	9.51	8.73	4.33	5.1	5.47	3.66	3.78	4.86
**G08**	1.03	0.8	0.49	1.38	1.7	1.62	0.28	0.54	0.37	0.28	0.26	0.3	0.41	0.17	0.17	0.22	0.29	0.24
**G09**	4.8	3.54	0.94	5.82	5.08	6.66	0.99	1.53	1.16	1.43	1.22	1.22	1.4	0.42	0.33	0.52	0.62	1.04
**G10**	5.7	5.47	1.46	6.09	6.52	8.24	1.49	1.93	2.08	2.23	3.84	3	3.06	1.71	1.29	1.21	1.29	3.9
**G11**	11	3.85	3.3	11.3	20.6	16.5	6.58	7.5	11.6	13.6	17.7	15.2	11.3	11	8.92	7.31	7.06	12.5
**G12**	2.74	1.54	1.8	2.9	5.96	2.6	4.43	1.95	2.75	2.98	5.36	4.27	2.52	4.22	3.24	2.34	2.59	5
**G13**	1.19	0.52	0.23	0.57	1.28	1.29	0.51	1.5	1.51	0.88	0.67	0.86	1.36	0.56	0.57	0.68	0.75	0.79
**G14**	3.07	1.74	0.83	2.55	7.81	5.33	2.29	3.88	2.74	1.8	1.58	1.59	1.91	1.1	1.16	1.24	1.25	1.36
**G15**	2.69	1.28	1.33	4.12	10.1	3.51	3.59	2.06	1.33	1	0.87	0.84	1.33	0.75	0.83	0.9	0.89	1.27
**G16**	9.98	7.76	2.02	3.82	7.97	3.73	6.12	5.54	4.91	4.4	4.01	4.52	5.85	5.29	6.08	7.16	7.88	9.15
**G17**	6.27	2.54	2.13	2.75	2.81	3.61	6.98	4.59	5.47	5.67	5.35	5.7	5.32	6.94	7.92	8.76	9.37	7.54
**G18**	3.33	1.47	2.54	1.99	1.98	3.22	6.49	3.72	4.18	4.76	4.79	5.52	6.74	5.95	6.34	7.1	6.6	5.04
**G19**	4.27	1.22	4.43	2.61	1.5	2.81	4.31	3.86	3.93	4.84	5.63	6.19	5.13	7.15	5.65	4.74	4.81	4.01
**G20**	2.9	1.34	1	1.68	3.55	2.19	2.96	3	1.75	1.38	1.46	1.44	1.52	1.32	1.44	1.37	1.26	1.34
**G21**	5.3	3.35	1.55	2.02	2.18	2.72	3.25	4.55	3.65	3.56	2.38	2.61	3.34	2.99	2.93	3.67	4.04	3.33
**G22**	4.3	2.41	1.78	2.17	1.83	2.03	4.87	3.55	4.48	4.94	4.41	4.29	3.55	6.25	5.62	5.77	6.1	4.88
**G23**	6.68	3	2.23	2.27	3.25	4.22	6.73	18	13.8	11.9	9.59	10.8	13.9	8.58	11.1	11.7	11.4	12
**G24**	2.98	1.28	2.38	2.07	1.68	1.72	5.02	3.89	3.42	4.21	3.57	3.42	4.11	3.88	4.34	4.16	4.12	3.56
**G25**	3.91	2.44	6.64	2.95	1.33	2.22	5.73	7.06	8.64	8.85	7.79	8.62	9.64	9.11	10.4	12	10.3	8.25
**G26**	2.17	0.86	2.54	1.52	0.77	1.52	2.88	3.83	3.54	2.87	2.2	2.15	2.04	3.02	2.89	3.13	3.27	2.31
**G27**	5.66	1.99	5.62	4.11	2.29	4.07	6.86	6.91	6.66	5.46	5.25	5.03	4.2	7.58	6.35	6.25	6.27	4.82
**G28**	1.8	0.47	1.23	1.51	1.44	2.45	2.05	2.81	2.23	1.99	1.62	1.7	2.24	1.55	1.59	1.46	1.53	1.72
**G29**	2.74	1.42	6.83	7.89	6.41	6.75	6.07	4	3.92	3.46	2.97	2.76	3	3.69	3.64	3.51	3.73	4
**G30**	1.3	1.85	16.3	6.98	2.42	1.79	3.45	1.98	1.73	1.69	1.31	1.25	1.14	1.83	1.74	1.78	1.78	1.51
**G31**	1.41	0.95	7.52	4.61	1.48	1.86	3.25	2.61	2.52	1.46	1.44	1.31	1.21	2.27	1.95	1.99	1.8	1.57

Day	41	45	48	50	52	55	57	62	64	66	69	71	73	76	83	90	92	94	97
**G01**	0.01	0.00	0.01	0.01	0.01	0.01	0.01	0.04	0.01	0.02	0.01	0.01	0.01	0.02	0.01	0.02	0.01	0.01	0.01
**G02**	0.01	0.01	0.00	0.00	0.01	0.01	0.01	0.06	0.04	0.03	0.03	0.02	0.02	0.04	0.03	0.03	0.04	0.02	0.04
**G03**	0.04	0.05	0.05	0.02	0.04	0.05	0.12	0.21	0.14	0.14	0.03	0.04	0.04	0.04	0.04	0.07	0.04	0.04	0.02
**G04**	0.07	0.11	0.1	0.06	0.09	0.11	0.16	0.19	0.23	0.38	0.05	0.08	0.07	0.04	0.12	0.12	0.07	0.13	0.05
**G05**	0.32	0.43	0.24	0.25	0.29	0.27	0.6	1.2	1.59	1.87	0.25	0.46	0.44	0.2	0.86	0.45	0.27	0.69	0.38
**G06**	0.52	0.45	0.21	0.34	0.58	0.66	0.69	0.45	0.27	0.4	0.16	0.19	0.16	0.25	0.32	0.25	0.26	0.22	0.15
**G07**	5.05	3.79	2.02	3.1	3.93	5.21	3.95	1.8	1.37	2.22	0.63	0.85	0.81	0.5	1.23	1.13	0.97	0.86	1.16
**G08**	0.3	0.37	0.33	0.25	0.35	0.38	0.59	1.11	1.1	0.96	0.32	0.33	0.36	0.58	0.47	0.32	0.27	0.36	0.18
**G09**	2	3.06	2.09	1.68	1.76	2.07	5.42	7.38	9.36	7.73	1.8	2.67	2.32	0.99	1.73	1.51	1.14	2.22	1.31
**G10**	5.9	7.6	4.85	4.98	7.18	8.86	11.9	3.54	3.38	3.07	2.06	2.32	1.93	1.13	1.55	2.1	1.88	1.18	1.33
**G11**	14.4	13.8	11.3	11.3	13.2	16.7	13.7	9.04	10.1	9.75	8.75	10.5	9.43	3.57	10.7	6.43	6.55	5.92	7.66
**G12**	5.79	5.92	5.53	6.07	7.73	8.37	5.24	1.81	1.65	1.7	3.64	4.68	2.93	1.31	5.74	2.92	3.35	1.71	1.74
**G13**	0.66	0.64	0.65	0.52	0.5	0.46	0.67	1.35	1.34	1.09	0.8	0.72	0.76	1.12	0.78	0.54	0.59	0.7	0.49
**G14**	1.3	1.4	1.36	1.18	1.24	1.18	1.6	2.37	2.48	1.82	1.76	1.59	1.75	3.75	1.89	1.17	1.23	1.32	1.04
**G15**	1.88	2.53	2.7	2.03	2.17	2.78	2.72	2.85	2.67	2.55	4.55	4.42	4.2	2.7	4.82	2.38	2.93	2.09	1.61
**G16**	12.2	14.4	17.2	17.9	16.2	13	11.9	15.2	18.3	17.1	31	32.9	32.9	12.9	34.5	23.9	31.3	29.1	20.2
**G17**	7.9	8.09	10.7	11.1	12.2	9.51	8.25	4.24	4.9	4.53	7.51	8.43	8.67	6.45	10	8.92	10.1	9.91	8.91
**G18**	4.04	3.08	3.57	3.75	4.16	4.08	3.55	2.21	1.88	2.14	2.31	1.99	2.36	3.61	2.75	3.52	3.19	3.07	3.19
**G19**	3.92	3.83	2.92	3.4	3.03	3.27	3.62	4.36	3.33	4.66	3.36	3.35	2.59	4	2.27	4.93	2.9	2.77	3.86
**G20**	1.5	1.59	1.9	1.64	1.49	1.52	1.6	4.55	4.4	4.28	4.41	3.71	5.05	9.74	3.72	3	3.36	4.47	4.64
**G21**	3.47	3.57	4.72	4.29	4.03	3.85	4.45	7.35	8.53	7.6	7.64	7.4	10.5	11.4	6.33	8.93	9.96	14.4	15.2
**G22**	5.19	4.45	4.87	4.06	4.25	4.08	4.02	3.29	3.7	3.75	3.1	2.99	3.24	5.44	2.59	4.06	3.45	3.98	5.77
**G23**	10.1	9.42	9.7	8.81	8.43	6.73	8.51	10.5	9.78	8.16	6.87	6.04	6.26	7.34	6.45	4.89	5.85	5.91	5.39
**G24**	3.22	2.99	3.73	3.49	3.01	2.69	2.66	2.62	2.63	2.52	2.3	1.97	1.92	3.35	1.88	2.53	2.23	2.16	2.89
**G25**	5.99	5.3	5.85	4.86	4.36	4.29	3.79	4.57	3.98	4.64	4.29	2.91	2.71	6.31	2.24	4.32	3.66	3.61	4.6
**G26**	1.81	1.84	1.82	1.92	1.69	1.73	1.5	2.17	2.1	2.5	2.97	2.51	2.13	2.93	1.84	3.35	2.49	2.84	3.5
**G27**	4.79	5.04	4.88	5.59	5.29	5.07	4.84	5.25	5.03	6.93	6.41	6.72	6.49	7.52	5.6	8.96	6.7	6.57	8.25
**G28**	1.65	1.65	1.71	1.51	1.28	1.24	1.36	1.91	2.05	1.69	1.82	1.63	1.75	2.07	1.91	1.36	1.73	1.69	1.29
**G29**	3.59	3.09	3.38	3.06	2.29	2.95	2.2	2.95	2.31	2.56	2.5	2.31	2.31	3.54	2.32	3.13	3.4	2.94	3.11
**G30**	1.18	1.07	0.94	1.01	0.69	1.09	0.77	1.41	1.1	1.43	1.04	0.82	0.71	2.07	0.59	1.68	1.36	1.18	1.85
**G31**	1.24	0.9	1.01	1.14	1.06	1.06	0.76	1.23	1.01	1.74	1.21	1.07	0.69	1.25	1.21	1.96	1.51	1.04	1.84

Day	99	101	104	106	108	111	115	118	120	122	125	127	129	132	134	136	139	141	146
**G01**	0.01	0.03	0.02	0.01	0.02	0.01	0.01	0.02	0.02	0.02	0.03	0.01	0.01	0.01	0.01	0.01	0.01	0.01	0.01
**G02**	0.01	0.02	0.02	0.02	0.02	0.02	0.01	0.01	0.01	0.02	0.02	0.03	0.01	0.01	0.01	0.01	0.01	0.01	0.02
**G03**	0.05	0.04	0.05	0.04	0.06	0.04	0.03	0.12	0.1	0.09	0.14	0.15	0.1	0.06	0.05	0.07	0.06	0.04	0.11
**G04**	0.11	0.14	0.14	0.07	0.23	0.08	0.07	0.24	0.24	0.2	0.27	0.27	0.14	0.1	0.13	0.12	0.09	0.05	0.11
**G05**	0.66	0.91	1.02	0.48	0.87	0.41	0.36	1.34	1.15	1.16	1.93	1.58	0.73	0.62	0.74	1.26	0.69	0.35	0.8
**G06**	0.18	0.22	0.26	0.2	0.2	0.15	0.12	0.3	0.38	0.47	0.64	0.49	0.26	0.27	0.29	0.59	0.31	0.26	0.57
**G07**	0.93	1.05	1.53	1.04	1.26	0.9	0.85	2.24	3.59	4.54	7.69	4.44	2.71	4.3	3.33	5.32	2.66	2.16	6.68
**G08**	0.41	0.41	0.49	0.3	0.47	0.3	0.3	0.86	0.71	0.7	0.96	1.73	1.5	0.53	0.73	0.54	0.4	0.33	0.82
**G09**	1.96	2.31	2.81	2.15	3.56	2.52	2.25	7.8	6.35	6.39	7.18	8.27	7.27	4.64	4.33	3.93	2.53	1.61	5.75
**G10**	2.38	2.51	3.21	2.22	2.18	1.73	1.79	4.6	4.27	5.07	5.2	4.05	2.83	3.21	2.57	3.82	2.31	2.37	8.57
**G11**	9.57	9.36	10.9	8.94	9.02	8.44	8.46	14.7	18.5	19.9	21.6	16.5	15.4	18.4	11.7	12.2	6.93	6.89	15.9
**G12**	2.42	1.99	2.72	2.28	1.67	1.61	1.88	2.12	2.1	3.14	3.42	2.25	1.78	2.38	1.6	2.14	1.32	2.07	4.08
**G13**	0.84	0.8	0.87	0.6	0.69	0.59	0.75	1.14	1.05	1.21	1.3	1.6	1.3	0.73	1.08	0.81	0.69	0.74	0.97
**G14**	1.97	1.75	1.88	1.31	1.69	1.43	1.69	2.6	2.11	2.47	2.6	3.9	5.39	2.45	2.37	1.86	1.56	1.52	2.26
**G15**	3.14	2.45	2.69	2.53	3.13	2.95	3.33	3.9	3.32	4.08	2.6	3.58	6.33	5.07	2.48	1.81	1.7	2.38	3.81
**G16**	19.2	15.8	17.7	17.2	15.6	16.1	14	8.69	7.71	8.61	5.92	6.14	8.89	11.2	7.08	8.29	9.78	6.75	4.08
**G17**	7.44	7.05	7.3	7.85	6.72	7.16	5.45	5.06	4.82	5.17	5.3	4.8	4.88	6.18	8.86	9.82	8.57	5.21	3.76
**G18**	3.04	3.05	2.81	2.77	2.25	2.44	2.11	2.44	2.27	2.17	2.07	2.13	2.18	2.3	2.48	2.66	2.27	2.17	1.94
**G19**	4.09	4.62	3.91	4.48	6.34	5.4	4.67	5.86	5.6	5.96	5.36	6.04	5.47	5.54	5.48	5.54	5.73	5.47	3.24
**G20**	5.57	4.4	4.5	3.65	3.56	3.48	3.66	2.41	1.77	1.99	1.62	1.7	1.91	2.07	1.55	1.68	2.03	2.41	1.84
**G21**	11.5	10.7	10.3	12	8.71	11.4	14.4	6.08	5.93	5.12	4.2	5.18	5.01	5.58	7.58	8.34	13	11	4.65
**G22**	5.45	5.78	5.18	6.13	5.06	5.07	5.92	5.71	5.51	5.42	6.28	6.1	7.84	9.39	9	10	12.2	10.9	7.53
**G23**	5.82	5.68	6.05	5.16	4.31	5.14	6.76	5.85	6.25	6.16	5.85	6.99	5.73	5.37	7.54	5.3	5.05	7.52	5.6
**G24**	2.5	2.74	2.49	3.01	2.64	3.13	4.33	3.48	3.91	3.14	3.25	4.33	5.13	5.02	5	3.98	5.6	6.57	3.95
**G25**	4.64	4.84	3.82	4.57	4.43	5.26	4.42	3.26	3.37	2.51	2.04	2.43	2.48	2.51	3.91	3.06	4.02	5.83	3.79
**G26**	3.82	4.12	3.25	3.72	3.65	4.49	3.9	3.36	2.73	1.67	1.33	1.55	1.63	1.66	2.22	1.82	2.51	3.09	1.87
**G27**	8.13	9.4	7.7	9.21	11.4	10.3	9.59	9.2	7.22	6.06	5.02	5.31	5.62	6.43	7.26	7.4	8.27	8.69	4.87
**G28**	1.62	1.62	1.8	1.39	1.57	1.62	2.01	1.91	2.18	2	1.98	2.29	2.12	1.55	2.25	1.69	1.87	1.78	1.85
**G29**	2.52	3.05	2.98	3.32	3.25	3.78	4.2	2.88	3.79	2.93	2.46	2.77	3.17	2.66	3.33	2.95	3.72	4.43	3.53
**G30**	1.39	1.64	1.24	1.94	2.14	2.4	1.97	1.16	1.5	1.03	0.8	0.83	1.12	1.03	1.12	1.2	1.61	2.36	1.71
**G31**	1.44	1.82	1.44	2.19	3.02	2.19	1.91	1.42	1.21	0.94	1.01	1.06	1.18	1.16	1.32	1.32	1.93	2.77	1.78

Day	148	150	153	155	157	160	162	164	167	169	175	181	183	185	188	190	195	197	199
G01	0.01	0.02	0.02	0.02	0.03	0.02	0.02	0.01	0.01	0.01	0.02	0.03	0.04	0.04	0.04	0.03	0.05	0.05	0.05
G02	0.01	0.02	0.01	0.01	0.01	0.01	0.03	0.02	0.02	0.01	0.01	0.02	0.02	0.04	0.03	0.03	0.04	0.04	0.04
G03	0.23	0.22	0.19	0.28	0.38	0.41	0.51	0.3	0.2	0.22	0.2	0.33	0.39	0.24	0.16	0.13	0.17	0.22	0.23
G04	0.24	0.25	0.22	0.33	0.35	0.27	0.43	0.25	0.21	0.19	0.19	0.28	0.37	0.4	0.27	0.24	0.28	0.39	0.43
G05	1.85	1.89	1.9	3.7	2.42	1.5	4.18	2.9	2.2	1.85	1.76	2.94	3.69	4.91	2.98	1.66	2.21	4.78	3.92
G06	1.01	1.07	0.62	0.68	0.89	0.49	0.83	0.87	0.81	0.73	0.56	0.55	0.69	1.1	1.23	1.21	1.73	1.59	1.49
G07	8.38	8.24	7.78	4.83	5.32	2.53	5.03	5.44	5.53	4.82	3.82	4.05	4.57	7.17	7.19	6.24	8.97	11	10.3
G08	1.5	1.24	1.24	1.26	1.49	1.85	2.08	1.56	1.32	1.24	1.01	1.36	1.4	1.01	0.95	0.83	1.2	1.29	1.79
G09	12.2	9.61	9.55	14.5	11.3	11.4	10.9	8.3	6.13	6.44	6.1	8.45	7.73	5.5	3.57	2.73	2.77	3.86	3.27
G10	9.15	10.5	5.92	6.39	10.1	8.28	4.94	5.46	3.99	3.94	3.78	3.88	3.92	2.68	3.18	4.35	4.39	3.51	3.03
G11	18.5	15.1	17.2	13.1	11.2	8.66	8.5	7.81	7.87	7.23	7.23	7.97	7.62	8.08	7.81	8.81	9.05	10.1	9.12
G12	2.83	2.96	1.97	1.53	2.06	1.82	1.48	1.23	1.38	1.15	1.12	1.16	1.46	1.7	1.87	3.21	2.61	2.44	2.61
G13	1.45	1.09	1.58	1.26	1.45	1.53	2.22	1.75	2.01	1.78	1.44	1.87	1.87	1.36	1.47	1.47	1.88	1.78	2.2
G14	3.34	2.42	3.31	2.9	2.88	3.42	3.96	4.37	5.35	4.65	4.26	4.62	4.54	4.22	4.31	4.45	5.82	5.33	6.23
G15	2.51	2.22	1.91	2.11	2.31	3.23	1.82	2.1	2.16	1.93	2.17	2.45	2.42	2.33	2.45	2.69	3.14	3.1	3.25
G16	3.97	4.97	9.37	9.99	10.2	8.07	6.23	5.52	5.59	5.25	6.09	7.59	8.36	7.75	7.93	7.72	7.79	7.25	6.14
G17	3.52	6.11	7.07	6.81	6.89	6.63	7.23	7.36	7.56	8.26	8.59	8.27	7.9	8.37	7.04	6.41	5.76	5.64	5.86
G18	1.78	2.74	2.57	2.81	3.14	3.94	4.47	4.48	4.71	4.15	4.26	3.79	4.15	4.34	4.96	4.1	4.8	4.74	4.28
G19	3.57	4.8	4.11	4.35	3.8	4.54	4.01	4.88	4.29	3.93	4.37	3.89	4.43	6.36	4.92	5.07	5.73	5.21	5.32
G20	1.65	1.67	2.18	2.36	2.32	2.4	2.16	2.67	2.53	2.57	2.79	3.08	2.77	2.75	3.35	3.63	3.5	3.34	3.36
G21	3.53	4.17	5.34	6.16	5.37	4.68	3.41	4.23	4.23	4.5	4.7	4.92	4.47	4.27	4.61	4.3	3.8	3.94	3.61
G22	4.49	6.1	3.67	5.48	4.36	4.75	4.01	5.34	6.33	7.93	7.52	5.16	4.88	4.44	4.57	4.33	2.6	2.02	1.97
G23	5.72	4.52	6.13	3.97	4.79	5.62	7.19	7.07	7.93	8.25	7.55	7.42	6.96	5.97	7.31	8.39	6.78	7.59	7.34
G24	3.03	2.17	2.38	2.2	2.27	2.74	3.12	3.68	4.54	4.9	4.57	3.94	4	3.52	4.01	4.24	3.13	3.26	2.98
G25	2.18	2.21	2.01	1.76	1.94	2.47	2.32	2.47	2.76	3.11	3.99	3.47	3.14	2.85	3.64	3.98	2.94	2.62	2.47
G26	1.32	1.45	1.21	1.28	1.22	1.56	1.45	1.73	1.73	1.81	2.78	2.46	2.5	2.51	2.61	2.5	2.27	1.79	1.84
G27	3.53	4.38	3.56	4.84	4.58	5.25	4.71	5.57	4.8	5.03	6.77	5.79	6.58	7.44	7.46	6.63	6.37	5.49	4.89
G28	2.09	1.56	2.13	1.62	2	2.39	2.89	2.6	2.81	2.64	2.38	2.62	2.41	1.82	2.16	2.34	2.23	2	2.49
G29	2.48	2.09	1.9	1.69	1.9	2.59	1.81	2.37	2.68	2.8	2.98	2.63	2.02	1.94	2.32	2.53	1.85	1.57	1.72
G30	0.88	0.86	0.58	0.56	0.55	0.76	0.39	0.58	0.79	0.83	0.86	0.82	0.63	0.68	0.77	0.8	0.6	0.44	0.45
G31	0.99	0.93	0.61	0.83	0.81	0.86	0.75	1.01	1.05	1.59	2.12	1.48	1.66	1.58	1.89	1.64	1.05	1.02	0.72

Day	202	204	206	209	211	216	218	220	225	227	230	232	234	237	239	241
**G01**	0.09	0.15	0.14	0.18	0.15	0.15	0.14	0.14	0.24	0.25	0.36	0.45	0.59	1.46	1.01	0.95
**G02**	0.05	0.08	0.10	0.14	0.15	0.16	0.10	0.13	0.14	0.25	0.56	0.84	0.82	3.07	2.69	2.07
**G03**	0.29	0.43	0.36	0.52	0.37	0.38	0.43	0.37	0.46	0.6	1.09	1.03	0.89	1.12	0.71	0.97
**G04**	0.64	1.06	0.62	0.94	0.62	0.57	0.84	0.52	1.25	1.41	1.63	1.55	1.53	2.21	0.97	1.14
**G05**	5.35	7.78	5.97	6.14	4.98	4.28	3.95	4.1	5.52	5.08	4.61	4.26	2.94	4.53	4.32	6.36
**G06**	1.44	1.64	1.65	1.89	1.78	2.17	2.81	3.61	5.47	7.94	9.95	12.1	10.5	11.4	10.1	9.68
**G07**	10.2	10.3	11.3	12.7	12.4	14.4	16.5	19.7	24.5	22.9	15.8	13.5	12.3	10.5	12.6	10.3
**G08**	1.76	1.64	1.48	1.85	1.65	1.55	1.82	1.82	1.96	1.92	2.58	3.1	3.06	3.21	3.15	3.6
**G09**	3.72	4.73	3.58	3.45	3.23	3.46	3.99	3.74	6.33	8.16	11.6	11.2	7.85	10.3	11.4	13.7
**G10**	2.86	2.95	2.92	3.44	4.15	8.85	10.5	12.6	16.1	19.2	21.4	22.1	23.5	21.5	22.9	19.8
**G11**	8.95	10	9.75	10.5	10.8	13.3	13.2	13.2	15	12.3	10.9	9.06	10.1	7.66	7.95	5.53
**G12**	1.99	1.98	2.16	2.59	2.54	3.99	3.28	2.98	2.09	1.73	1.3	1.08	1.4	0.91	1.04	0.75
**G13**	2.28	2.11	2.07	2.05	2.07	1.92	1.84	1.69	1.46	1.56	1.67	1.63	1.63	1.76	1.4	1.58
**G14**	6.03	5.51	6.31	6.36	7.04	6.46	6.61	5.79	4.24	3.83	3.63	3.84	3.87	3.8	3.34	3.96
**G15**	2.93	2.66	2.77	2.74	2.76	3.26	2.88	2.52	1.5	1.4	2.02	2.65	3.95	3.25	2.74	2.71
**G16**	5.99	5.55	5.51	5.26	5.06	4.2	3.72	3.49	2.45	2.12	1.56	1.43	1.63	1.73	1.66	1.98
**G17**	7.03	6.47	6.01	6.05	5.83	5.3	4.55	4.46	2.55	1.55	0.88	0.68	0.68	0.57	0.61	0.55
**G18**	5.25	4.81	4.66	4.46	4.41	4.24	3.64	3.17	2.04	1.04	0.74	0.62	0.62	0.55	0.6	0.59
**G19**	5.57	5.84	5.45	5.11	4.87	3.77	3.43	3.49	2.25	2.04	2.15	2.35	2.47	1.84	2.31	2.71
**G20**	2.81	2.45	3.15	3.41	4.03	4.4	4.66	3.73	3.38	3.67	4.11	4.23	4.5	3.84	3.49	3.77
**G21**	2.93	2.89	3.37	3.36	3.57	2.81	2.98	2.61	2.12	1.49	1.28	1.02	1.19	1.12	0.96	0.99
**G22**	2	1.47	1.58	1.37	1.55	1.51	1.15	0.95	0.66	0.49	0.5	0.48	0.53	0.47	0.43	0.39
**G23**	7.58	6.81	7.28	5.68	5.98	4.5	4.28	3.61	2.72	1.78	1.74	1.52	1.68	1.47	1.23	1.23
**G24**	2.76	2.59	2.95	2.17	2.29	1.5	1.45	1.19	0.66	0.42	0.4	0.29	0.38	0.33	0.33	0.3
**G25**	2.53	2.48	2.33	1.88	1.97	1.43	1.64	1.42	0.92	0.85	0.96	0.9	0.92	0.85	0.8	0.92
**G26**	1.64	1.57	1.54	1.49	1.66	1.36	1.52	1.34	0.93	0.84	0.94	0.99	1.11	1.27	1.08	1.12
**G27**	4.54	4.46	4.27	4.15	4.32	3.54	3.39	3.22	2.2	1.91	1.93	2.03	2.37	2.14	2.38	2.66
**G28**	2.35	2.05	2.09	1.87	1.9	1.49	1.37	1.2	0.96	0.91	0.98	0.9	1.03	0.95	0.9	0.91
**G29**	1.79	1.47	1.48	1.18	1.25	0.9	0.82	0.72	0.47	0.41	0.47	0.4	0.55	0.47	0.52	0.44
**G30**	0.43	0.4	0.39	0.29	0.27	0.2	0.21	0.2	0.12	0.12	0.15	0.13	0.17	0.15	0.16	0.2
**G31**	0.73	0.61	0.81	0.92	0.86	0.86	0.72	0.51	0.44	0.43	0.48	0.5	0.61	0.53	0.47	0.55

S11: Correlation analysis

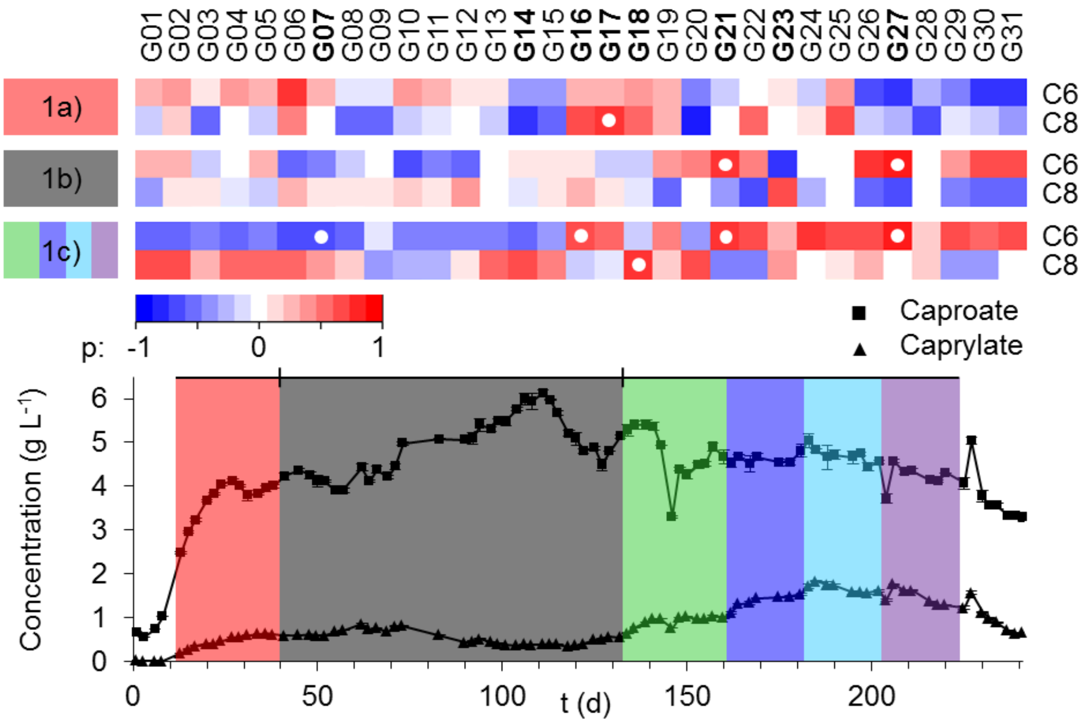

Figure S11: Spearman's rank order correlation was applied to test for links between the relative cell abundance values of all 31 sub-communities and caproate (C6) and caprylate (C8) concentrations. Correlation was calculated in three distinct periods defined by the main experimental periods: 1a) stage 2 - TAN-shortage ●, 1b) stage 3 - consolidation ● and 1c) stages 4 - pH 5.75 ●, 5 - pH 6.0 ●, 6 - pH 6.25 ● and 7 - pH 6.5 ●. Correlation strength (r_S), significance (p) and corrected significance (p_{BH}) are provided in Additional file 1 Table 1 S11. Sub-communities chosen for sorting are marked in bold and additionally provided in Additional file 1 Table 3 S11. The strong correlations (r_S > |±0.7|) these sub-communities were chosen for are marked with a white dot.

Table 1 S11: Spearman's correlation coefficient (r_S), significance (p) and Benjamini Hochberg corrected significance (p_{BH}) values of the relative cell abundances in all 31 gates (Additional file 1 S8) correlating with the concentration of caproate and caprylate. The correlation analysis was performed in three distinct periods defined by the main experimental periods 1a) stage 2 from day 13 to 40, 1b) stage 3 from day 41 to 133 and 1c) stages 4 to 7 from day 134 to 224. The gates are ordered according to their correlation strength r_S. The gates correlating stonger than |±0.7| are shaded (caproate: ● negative, ● positive correlation, caprylate:● negative, ● positive).

1a) Stage 2: days 13 - 40

	Caproate				Caprylate		
**Gates**	**Strength r_S**	**Significance p**	**p_{BH} corrected**	**Gates**	**Strength r_S**	**Significance p**	**p_{BH} corrected**
**G27**	-0.79860	0.00184	0.02379	**G20**	-0.83916	0.00064	0.01228
**G31**	-0.78109	0.00271	0.02806	**G14**	-0.80420	0.00161	0.02284
**G30**	-0.72983	0.00705	0.05295	**G28**	-0.69930	0.01137	0.07244
**G26**	-0.62937	0.02832	0.12691	**G09**	-0.58042	0.04786	0.16866
**G20**	-0.47552	0.11818	0.28535	**G15**	-0.57443	0.05077	0.17073
**G29**	-0.44056	0.15174	0.33655	**G08**	-0.56440	0.05592	0.18259
**G14**	-0.39860	0.19934	0.40274	**G03**	-0.55029	0.06376	0.19737
**G15**	-0.35377	0.25927	0.47135	**G31**	-0.35377	0.25927	0.47135
**G28**	-0.27972	0.37857	0.58477	**G27**	-0.31173	0.32394	0.53209
**G24**	-0.22767	0.47668	0.68213	**G13**	-0.23776	0.45680	0.66773
**G21**	-0.22378	0.48445	0.68426	**G30**	-0.22456	0.48288	0.68426
**G08**	-0.10582	0.74342	0.85511	**G05**	-0.21678	0.49856	0.68636
**G09**	-0.05594	0.86290	0.92738	**G01**	-0.21672	0.49868	0.68636
**G22**	0.00699	0.98279	0.98824	**G10**	-0.19580	0.54194	0.71190
**G13**	0.06993	0.82902	0.91101	**G26**	-0.14685	0.64880	0.79476
**G03**	0.07055	0.82753	0.91101	**G29**	-0.11888	0.71288	0.83387
**G23**	0.12587	0.69668	0.82917	**G11**	-0.07692	0.81218	0.90529
**G12**	0.13287	0.68060	0.81717	**G23**	-0.02797	0.93123	0.97044
**G07**	0.24476	0.44326	0.65675	**G21**	-0.02098	0.94840	0.97527
**G17**	0.27972	0.37857	0.58477	**G04**	-0.01051	0.97414	0.98683
**G01**	0.28423	0.37060	0.58045	**G12**	0.00000	1.00000	1.00000
**G16**	0.29371	0.35415	0.56138	**G07**	0.02098	0.94840	0.97527
**G19**	0.29371	0.35415	0.56138	**G24**	0.07005	0.82873	0.91101
**G05**	0.30769	0.33059	0.53653	**G02**	0.18981	0.55461	0.72263
**G11**	0.32867	0.29690	0.50128	**G19**	0.25874	0.41677	0.63125
**G25**	0.37762	0.22621	0.43293	**G06**	0.49037	0.10555	0.27113
**G10**	0.38462	0.21702	0.42187	**G18**	0.58494	0.04574	0.16225
**G18**	0.41331	0.18172	0.38014	**G22**	0.59441	0.04152	0.15123
**G02**	0.41477	0.18003	0.37921	**G16**	0.62238	0.03068	0.12998
**G04**	0.42032	0.17369	0.37002	**G25**	0.68531	0.01391	0.08275
**G06**	0.78109	0.00271	0.02806	**G17**	0.72727	0.00736	0.05304

1b) Stage 3: days 41 - 133

	Caproate				Caprylate		
**Gates**	**Strength r_S**	**Significance p**	**p_{BH} corrected**	**Gates**	**Strength r_S**	**Significance p**	**p_{BH} corrected**
**G23**	-0.75693	0.00000	0.00001	**G27**	-0.68871	0.00002	0.00012
**G10**	-0.65800	0.00006	0.00035	**G22**	-0.67702	0.00003	0.00018
**G06**	-0.59867	0.00037	0.00183	**G30**	-0.57094	0.00080	0.00351
**G12**	-0.59018	0.00047	0.00216	**G31**	-0.56627	0.00090	0.00386
**G07**	-0.48785	0.00537	0.01705	**G26**	-0.56538	0.00092	0.00392
**G11**	-0.43996	0.01326	0.03579	**G19**	-0.56331	0.00097	0.00410
**G03**	-0.21529	0.24477	0.38463	**G29**	-0.52340	0.00252	0.00921
**G18**	-0.19560	0.29163	0.43684	**G21**	-0.37016	0.04039	0.09183
**G08**	-0.15577	0.40272	0.56010	**G01**	-0.34625	0.05637	0.12109
**G17**	-0.15363	0.40930	0.56780	**G24**	-0.31360	0.08580	0.16959
**G24**	-0.04175	0.82355	0.89595	**G05**	-0.19581	0.29112	0.43684
**G09**	-0.03669	0.84463	0.91057	**G04**	-0.11755	0.52883	0.69135
**G04**	-0.02323	0.90129	0.94832	**G18**	-0.08006	0.66857	0.79225
**G25**	-0.00323	0.98626	0.99172	**G14**	-0.06090	0.74485	0.84056
**G28**	0.04422	0.81327	0.89124	**G25**	-0.02843	0.87933	0.93606
**G13**	0.04760	0.79930	0.88190	**G28**	-0.02423	0.89705	0.94756
**G16**	0.06755	0.71804	0.82124	**G20**	0.00000	1.00000	1.00000
**G14**	0.09962	0.59391	0.74256	**G13**	0.01553	0.93392	0.96585
**G15**	0.11574	0.53525	0.69640	**G15**	0.05021	0.78853	0.87179
**G05**	0.23956	0.19428	0.32802	**G09**	0.05242	0.77943	0.86879
**G02**	0.24483	0.18436	0.31581	**G07**	0.06292	0.73667	0.83655
**G01**	0.27603	0.13281	0.23906	**G11**	0.07642	0.68284	0.80390
**G29**	0.41448	0.02043	0.05069	**G08**	0.09988	0.59293	0.74256
**G19**	0.42298	0.01775	0.04485	**G02**	0.10738	0.56533	0.72318
**G22**	0.44516	0.01209	0.03424	**G17**	0.11815	0.52674	0.69027
**G20**	0.47066	0.00754	0.02299	**G03**	0.12571	0.50042	0.66343
**G31**	0.63123	0.00014	0.00076	**G10**	0.21920	0.23611	0.37792
**G30**	0.63890	0.00011	0.00061	**G06**	0.23870	0.19594	0.32980
**G21**	0.74476	0.00000	0.00001	**G16**	0.28715	0.11728	0.21982
**G26**	0.75411	0.00000	0.00001	**G12**	0.33753	0.06331	0.13417
**G27**	0.84073	0.00000	0.00000	**G23**	0.65168	0.00007	0.00043

1 c) Stages 4 – 7: days 134 - 224

	Caproate				Caprylate		
Gates	Strength r_S	Significance p	p_{BH} corrected	Gates	Strength r_S	Significance p	p_{BH} corrected
**G07**	-0.70161	0.00001	0.00005	**G22**	-0.45710	0.00973	0.01707
**G06**	-0.65477	0.00006	0.00021	**G21**	-0.42968	0.01585	0.02667
**G08**	-0.58907	0.00049	0.00124	**G30**	-0.38636	0.03180	0.04857
**G14**	-0.57903	0.00064	0.00157	**G29**	-0.37185	0.03942	0.05856
**G01**	-0.56511	0.00092	0.00216	**G09**	-0.33992	0.06135	0.08621
**G04**	-0.56324	0.00097	0.00224	**G10**	-0.28352	0.12219	0.16013
**G02**	-0.53265	0.00204	0.00432	**G11**	-0.27886	0.12873	0.16755
**G11**	-0.52183	0.00261	0.00532	**G16**	-0.15161	0.41554	0.46959
**G05**	-0.50670	0.00363	0.00709	**G17**	-0.06271	0.73753	0.77154
**G03**	-0.47996	0.00629	0.01154	**G24**	-0.02964	0.87422	0.89392
**G13**	-0.47609	0.00678	0.01237	**G27**	0.00605	0.97424	0.98145
**G12**	-0.47303	0.00720	0.01296	**G31**	0.04579	0.80675	0.83275
**G10**	-0.44747	0.01160	0.02009	**G12**	0.09295	0.61894	0.65631
**G20**	-0.35310	0.05136	0.07408	**G25**	0.10950	0.55763	0.60346
**G15**	-0.34556	0.05690	0.08079	**G19**	0.15143	0.41612	0.46959
**G18**	-0.20565	0.26707	0.31172	**G08**	0.16542	0.37382	0.42450
**G09**	-0.06694	0.72052	0.75958	**G28**	0.21030	0.25614	0.30090
**G28**	0.22845	0.21642	0.26040	**G03**	0.25038	0.17430	0.21693
**G23**	0.30971	0.08998	0.12023	**G26**	0.25870	0.15996	0.20231
**G19**	0.45549	0.01003	0.01753	**G23**	0.34217	0.05954	0.08410
**G17**	0.52667	0.00234	0.00486	**G07**	0.41411	0.02056	0.03346
**G30**	0.59921	0.00037	0.00097	**G15**	0.44032	0.01318	0.02251
**G26**	0.62405	0.00018	0.00052	**G04**	0.53198	0.00207	0.00437
**G22**	0.64361	0.00009	0.00030	**G13**	0.53885	0.00176	0.00377
**G31**	0.66028	0.00005	0.00018	**G06**	0.53983	0.00172	0.00373
**G29**	0.66405	0.00005	0.00016	**G05**	0.60954	0.00027	0.00076
**G25**	0.67211	0.00003	0.00013	**G14**	0.65323	0.00007	0.00022
**G16**	0.68911	0.00002	0.00007	**G02**	0.65577	0.00006	0.00021
**G24**	0.71869	0.00001	0.00002	**G01**	0.66949	0.00004	0.00014
**G21**	0.73778	0.00000	0.00001	**G20**	0.69470	0.00001	0.00006
**G27**	0.81157	0.00000	0.00000	**G18**	0.74516	0.00000	0.00001

Table 2 S11: Spearman's correlation coefficient (r_S), significance (p) and Benjamini Hochberg corrected significance (p_{BH}) values of the relative cell abundances in all 31 gates (Additional file 1 S8) correlating with the concentration of caproate and caprylate. The correlation analysis was performed in three distinct periods defined by 2a) the initial caproate and caprylate increase from day 6 to day 45, 2c) the secondary caproate increase from day 57 to day 111 and 2c) the secondary caprylate increase from day 132 to day 185. The gates are ordered according to their correlation strength r_S. The gates correlating stonger than |±0.7| are shaded (caproate: ● negative, ● positive correlation, caprylate:● negative, ● positive).

2a) Inital C6/C8 increase: days 6 - 45

	Caproate				Caprylate		
**Gates**	**Strength r_S**	**Significance p**	**p_{BH} corrected**	**Gates**	**Strength r_S**	**Significance p**	**p_{BH} corrected**
**G30**	-0.83248	0.00006	0.00099	**G14**	-0.83824	0.00005	0.00086
**G31**	-0.73731	0.00112	0.00929	**G20**	-0.75000	0.00082	0.00726
**G29**	-0.64706	0.00674	0.03447	**G08**	-0.55097	0.02697	0.08767
**G14**	-0.58235	0.01793	0.06665	**G15**	-0.51803	0.03983	0.11434
**G20**	-0.47941	0.06024	0.15435	**G01**	-0.50599	0.04553	0.12379
**G15**	-0.33407	0.20602	0.35896	**G30**	-0.49594	0.05073	0.13442
**G13**	-0.26932	0.31313	0.48645	**G03**	-0.46382	0.07035	0.17139
**G01**	-0.25151	0.34740	0.52038	**G09**	-0.45882	0.07384	0.17829
**G27**	-0.24742	0.35554	0.52501	**G13**	-0.42237	0.10315	0.22602
**G08**	-0.23929	0.37207	0.54097	**G31**	-0.40912	0.11559	0.24697
**G02**	-0.20339	0.44994	0.60269	**G28**	-0.39441	0.13060	0.26290
**G26**	-0.18235	0.49907	0.64625	**G05**	-0.38999	0.13536	0.26948
**G07**	-0.16483	0.54185	0.69176	**G29**	-0.38235	0.14386	0.27926
**G28**	-0.15158	0.57521	0.71589	**G07**	-0.30611	0.24889	0.41315
**G09**	-0.10294	0.70441	0.80833	**G02**	-0.28887	0.27787	0.45232
**G03**	-0.04284	0.87484	0.94420	**G11**	-0.28824	0.27898	0.45278
**G05**	-0.03091	0.90954	0.95148	**G10**	-0.23235	0.38651	0.55165
**G24**	-0.01325	0.96117	0.98099	**G04**	-0.21355	0.42712	0.58360
**G11**	0.01765	0.94828	0.97699	**G06**	-0.00883	0.97411	0.98863
**G06**	0.05740	0.83279	0.90963	**G27**	0.06627	0.80734	0.89077
**G04**	0.09720	0.72025	0.81664	**G26**	0.12647	0.64069	0.76420
**G10**	0.12647	0.64069	0.76420	**G12**	0.13392	0.62097	0.75389
**G21**	0.18529	0.49206	0.64328	**G24**	0.22075	0.41131	0.57255
**G19**	0.21765	0.41809	0.57852	**G23**	0.27059	0.31076	0.48415
**G18**	0.24724	0.35591	0.52501	**G19**	0.27647	0.29994	0.47170
**G23**	0.27353	0.30532	0.47704	**G21**	0.31471	0.23516	0.39910
**G25**	0.27647	0.29994	0.47170	**G18**	0.44150	0.08689	0.20176
**G12**	0.28550	0.28377	0.45571	**G25**	0.51765	0.04000	0.11434
**G22**	0.31765	0.23057	0.39295	**G16**	0.60882	0.01232	0.05080
**G16**	0.50294	0.04706	0.12593	**G22**	0.66765	0.00471	0.02659
**G17**	0.59118	0.01588	0.06024	**G17**	0.84118	0.00004	0.00085

2b) Secondary C6 increase: days 55 - 111

	Caproate				Caprylate		
Gates	Strength r_S	Significance p	p_{BH} corrected	Gates	Strength r_S	Significance p	p_{BH} corrected
**G23**	-0.81404	0.00002	0.00033	**G26**	-0.79825	0.00004	0.00056
**G06**	-0.52947	0.01974	0.06942	**G22**	-0.77719	0.00009	0.00113
**G10**	-0.48596	0.03490	0.10299	**G27**	-0.77368	0.00010	0.00125
**G12**	-0.47193	0.04135	0.11818	**G29**	-0.69214	0.00102	0.00791
**G11**	-0.44932	0.05361	0.14560	**G31**	-0.66842	0.00176	0.01106
**G03**	-0.41440	0.07773	0.18687	**G30**	-0.59763	0.00689	0.03036
**G08**	-0.35997	0.13007	0.27293	**G21**	-0.55789	0.01306	0.05026
**G07**	-0.33802	0.15695	0.31132	**G19**	-0.46140	0.04676	0.13022
**G09**	-0.19123	0.43290	0.60517	**G25**	-0.41579	0.07664	0.18651
**G13**	-0.17113	0.48363	0.64471	**G18**	-0.41053	0.08083	0.19178
**G14**	-0.15364	0.53001	0.68630	**G24**	-0.34504	0.14796	0.30006
**G15**	-0.14737	0.54714	0.70513	**G01**	-0.23348	0.33604	0.50912
**G28**	-0.14330	0.55838	0.71792	**G17**	-0.09825	0.68905	0.81418
**G04**	-0.14254	0.56047	0.71891	**G05**	0.00965	0.96871	0.98421
**G02**	-0.09311	0.70461	0.82596	**G04**	0.04312	0.86088	0.91806
**G17**	0.01404	0.95452	0.97603	**G07**	0.05004	0.83878	0.91618
**G16**	0.03072	0.90066	0.94039	**G20**	0.13778	0.57378	0.72070
**G18**	0.04737	0.84730	0.91682	**G02**	0.17567	0.47191	0.64053
**G01**	0.05551	0.82144	0.90475	**G16**	0.18166	0.45669	0.62558
**G05**	0.06319	0.79720	0.88316	**G10**	0.22632	0.35150	0.52221
**G20**	0.13778	0.57378	0.72070	**G12**	0.26491	0.27305	0.44315
**G24**	0.18701	0.44332	0.61343	**G06**	0.26913	0.26520	0.43299
**G25**	0.36842	0.12065	0.26016	**G14**	0.27392	0.25645	0.42378
**G19**	0.42807	0.06749	0.16665	**G11**	0.28609	0.23507	0.39689
**G22**	0.59649	0.00702	0.03071	**G03**	0.29061	0.22744	0.38853
**G31**	0.63684	0.00337	0.01823	**G08**	0.29500	0.22017	0.38363
**G30**	0.64326	0.00297	0.01668	**G13**	0.29750	0.21610	0.38231
**G29**	0.67896	0.00139	0.00918	**G15**	0.29825	0.21489	0.38175
**G21**	0.74211	0.00027	0.00280	**G09**	0.30702	0.20105	0.36895
**G27**	0.83684	0.00001	0.00015	**G28**	0.36484	0.12458	0.26654
**G26**	0.86667	0.00000	0.00004	**G23**	0.78596	0.00007	0.00087

2c) Secondary C8 increase: days 132 - 185

	Caproate				Caprylate		
Gates	Strength r_S	Significance p	p_{BH} corrected	Gates	Strength r_S	Significance p	p_{BH} corrected
G09	-0.68246	0.00128	0.00663	G21	-0.61228	0.00533	0.01940
G10	-0.67691	0.00146	0.00738	G12	-0.54912	0.01489	0.04126
G07	-0.57193	0.01051	0.03188	G30	-0.49934	0.02951	0.06940
G06	-0.55048	0.01459	0.04079	G22	-0.45439	0.05065	0.10349
G08	-0.49055	0.03297	0.07496	G11	-0.43860	0.06032	0.11750
G13	-0.45958	0.04775	0.09904	G16	-0.37544	0.11320	0.19474
G14	-0.44035	0.05918	0.11612	G19	-0.31228	0.19304	0.29364
G05	-0.38772	0.10097	0.17592	G29	-0.31154	0.19416	0.29407
G01	-0.35211	0.13928	0.22819	G24	-0.18692	0.44352	0.57196
G04	-0.35046	0.14128	0.23066	G27	-0.12456	0.61140	0.72608
G11	-0.33333	0.16314	0.25562	G15	-0.00351	0.98863	0.99410
G03	-0.32133	0.17976	0.27689	G25	0.05968	0.80826	0.87407
G18	-0.30526	0.20377	0.30440	G10	0.07550	0.75868	0.84221
G28	-0.22632	0.35150	0.48270	G31	0.16945	0.48800	0.61013
G02	-0.19207	0.43084	0.56596	G26	0.20526	0.39922	0.53607
G20	-0.05088	0.83613	0.89182	G07	0.21404	0.37892	0.51517
G23	-0.04912	0.84171	0.89427	G09	0.24386	0.31437	0.44174
G12	-0.00702	0.97725	0.98814	G17	0.30702	0.20105	0.30117
G15	0.03860	0.87534	0.91395	G23	0.35965	0.13044	0.21887
G17	0.28421	0.23830	0.34803	G08	0.46857	0.04302	0.09167
G30	0.41509	0.07718	0.14368	G06	0.53995	0.01702	0.04531
G29	0.46336	0.04571	0.09658	G28	0.57193	0.01051	0.03188
G31	0.48727	0.03434	0.07743	G03	0.62248	0.00442	0.01667
G22	0.51228	0.02493	0.05993	G04	0.63680	0.00337	0.01364
G16	0.52632	0.02062	0.05186	G02	0.66961	0.00171	0.00836
G26	0.55088	0.01451	0.04079	G13	0.68893	0.00111	0.00587
G25	0.56428	0.01184	0.03477	G05	0.69825	0.00088	0.00510
G19	0.59298	0.00745	0.02513	G01	0.77113	0.00011	0.00097
G24	0.61343	0.00522	0.01914	G18	0.78246	0.00008	0.00070
G21	0.68246	0.00128	0.00663	G20	0.79649	0.00004	0.00045
G27	0.80000	0.00004	0.00040	G14	0.83333	0.00001	0.00013

Table 3 S11: Strength (r_S) and Benjamini Hochberg corrected significance (p_{BH}) of the relative cell abundances of the eight sorted gates (Additional file 1 S12) with the carboxylates caproate and caprylate. The gates that correlated stonger than |±0.7| and displayed more than 5% relative cell abundance have been chosen for cell sorting. Coefficients > |±0.7| are shaded (caproate: ● negative, ● positive correlation, caprylate:● negative, ● positive correlation).

	1) Experimental stages											
	1a) Stage 2: day 15 - 38				1b) Stage 3 day: 41 - 132				1c) Stages 4 - 7 day: 134 - 220			
	Caproate		Caprylate		Caproate		Caprylate		Caproate		Caprylate	
	r_S	p_{BH}	r_S	p_{BH}	r_S	p_{BH}	r_S	p_{BH}	r_S	p_{BH}	r_S	p_{BH}
**G07**	0.24	0.657	0.02	0.975	-0.49	0.017	0.06	0.837	-0.70	4.5×10^{-5}	0.41	0.033
**G14**	-0.40	0.403	-0.80	0.023	0.10	0.743	-0.06	0.841	-0.58	0.002	0.65	$2.2\text{x}10^{-4}$
**G16**	0.29	0.561	0.62	0.130	0.07	0.821	0.29	0.220	0.69	7.2×10^{-5}	-0.15	0.470
**G17**	0.28	0.585	0.73	0.053	-0.15	0.568	0.12	0.690	0.53	0.005	-0.06	0.772
**G18**	0.41	0.380	0.58	0.162	-0.20	0.437	-0.08	0.792	-0.21	0.312	0.75	8.2×10^{-6}
**G21**	-0.22	0.684	-0.02	0.975	0.74	1.3×10^{-5}	-0.37	0.092	0.74	1.2×10^{-5}	-0.43	0.027
**G23**	0.13	0.829	-0.03	0.970	-0.76	8.2×10^{-6}	0.65	0.000	0.31	0.120	0.34	0.084
**G27**	-0.80	0.024	-0.31	0.532	0.84	6.7×10^{-8}	-0.69	0.000	0.81	2.6×10^{-7}	0.01	0.981

	2) Product concentration inceases											
	2a) Inital C6 C8 increase: day 6 - 45				2b) Secondary C6 increase: day 55 - 111				2c) Secondary C8 increase: day 132 - 185			
	Caproate		Caprylate		Caproate		Caprylate		Caproate		Caprylate	
	r_S	p_{BH}	r_S	p_{BH}	r_S	p_{BH}	r_S	p_{BH}	r_S	p_{BH}	r_S	p_{BH}
**G07**	-0.16	0.692	-0.31	0.413	-0.34	0.311	0.05	0.916	-0.57	0.032	0.21	0.515
**G14**	-0.58	0.067	-0.84	0.001	-0.15	0.686	0.27	0.424	-0.44	0.116	0.83	1.3×10^{-4}
**G16**	0.50	0.126	0.61	0.051	0.03	0.940	0.18	0.626	0.53	0.052	-0.38	0.195
**G17**	0.59	0.060	0.84	0.001	0.01	0.976	-0.10	0.814	0.28	0.348	0.31	0.301
**G18**	0.25	0.525	0.44	0.202	0.05	0.917	-0.41	0.192	-0.31	0.304	0.78	0.001
**G21**	0.19	0.643	0.31	0.399	0.74	0.003	-0.56	0.050	0.68	0.007	-0.61	0.019
**G23**	0.27	0.477	0.27	0.484	-0.81	3.3×10^{-4}	0.79	0.001	-0.05	0.894	0.36	0.219
**G27**	-0.25	0.525	0.07	0.891	0.84	1.4×10^{-4}	-0.77	0.001	0.80	3.9×10^{-4}	-0.12	0.726

S12: Flow cytometric cell sorting

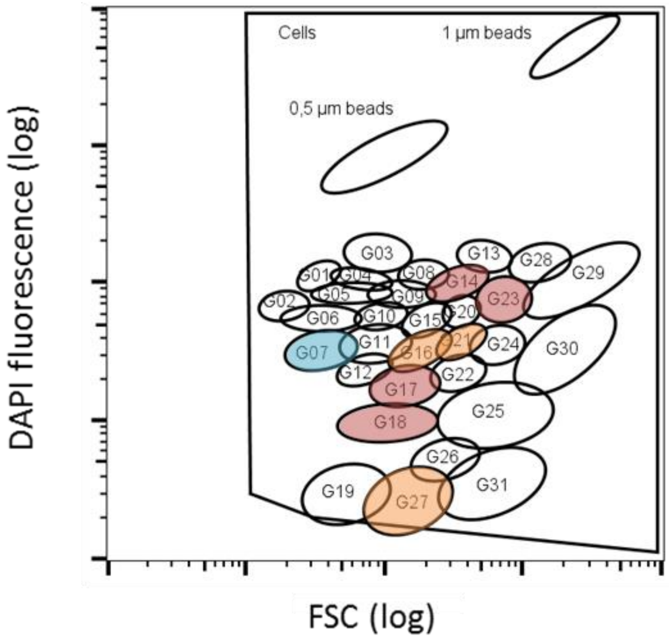

Figure 1 S12: Overview of sub-communities selected for cell sorting. They are characterised by strong, significant Spearman's correlations (r_S > |±0.7|, p < 0.5) between relative cell abundance and product concentrations (caproate: ● negative, ● positive correlation, caprylate:● negative, ● positive correlation). Correlation strength (r_S), significance (p) and corrected significance (p_{BH}) are given in Additional file 1 Table 1 S11.

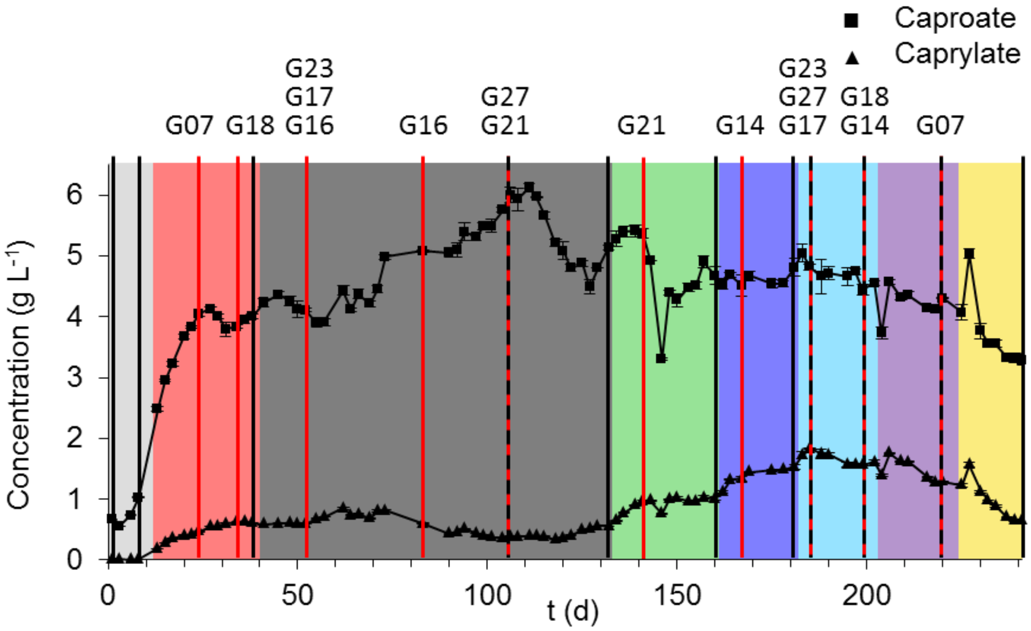

Figure 2 S12: Overview of sampling points analysed by MiSeq amplicon sequencing (details in Table S12). The whole community was analysed at eleven time points (inoculum day 1, end of each stage days 8, 38, 132, 160, 181, 202, 241, caproate and caprylate concentration peaks, days 106 and 185, time points are marked with black lines). Eight different sub-communities (G07, G14, G16, G17, G18, G21, G23, G27, position in gate in Figure 1 S12, time points marked with red lines) were analysed at two time points each.

Table S12: Whole community and sorted sub-community sampling points analysed by MiSeq amplicon sequencing. The time periods of the eight experimental stages are colour-coded: 1 - start-up ●, 2 - TAN-shortage ●, 3 - consolidation ●, 4 - pH 5.75 ●, 5 - pH 6.0 ●, 6 - pH 6.25 ●, 7 - pH 6.5 ● and 8 - pH 7 ●

**Whole communitys** WC #	**Stage / reason**	**Day**	**Rel. cell abundance** %
1	Inoculum	1	
2	1 - start-up	8	
3	2 - TAN-shortage	38	
4	3 - consolidation	132	
5	4 - pH 5.75	160	
6	5 - pH 6.0	181	
7	6 - pH 6.25	202	
8	7 - pH 6.5	220	
9	8 - pH 7.0	241	
10	C6 peak	106	
11	C8 peak	185	
**Sorted sub-communities**			
G07	high abundance in the start-up and pH 7.0 stage	24<br>220	8.73<br>19.7
G14	high abundance at C8 concentration peak	167<br>202	5.35<br>6.03
G16	very high overall abundance	52<br>83	16.2<br>34.5
G17	r_S>|±0.7|, p < 0.5 in all three correlation scenarios	52<br>185	12.2<br>8.37
G18	r_S >|±0.75|, p<0.5 in two correlation scenarios	34<br>202	7.1<br>5.25
G21		106<br>141	12<br>11
G23	high abundance at C6, C8 concentration peaks	52<br>185	10.5<br>5.97
G27		106<br>185	9.21<br>7.44

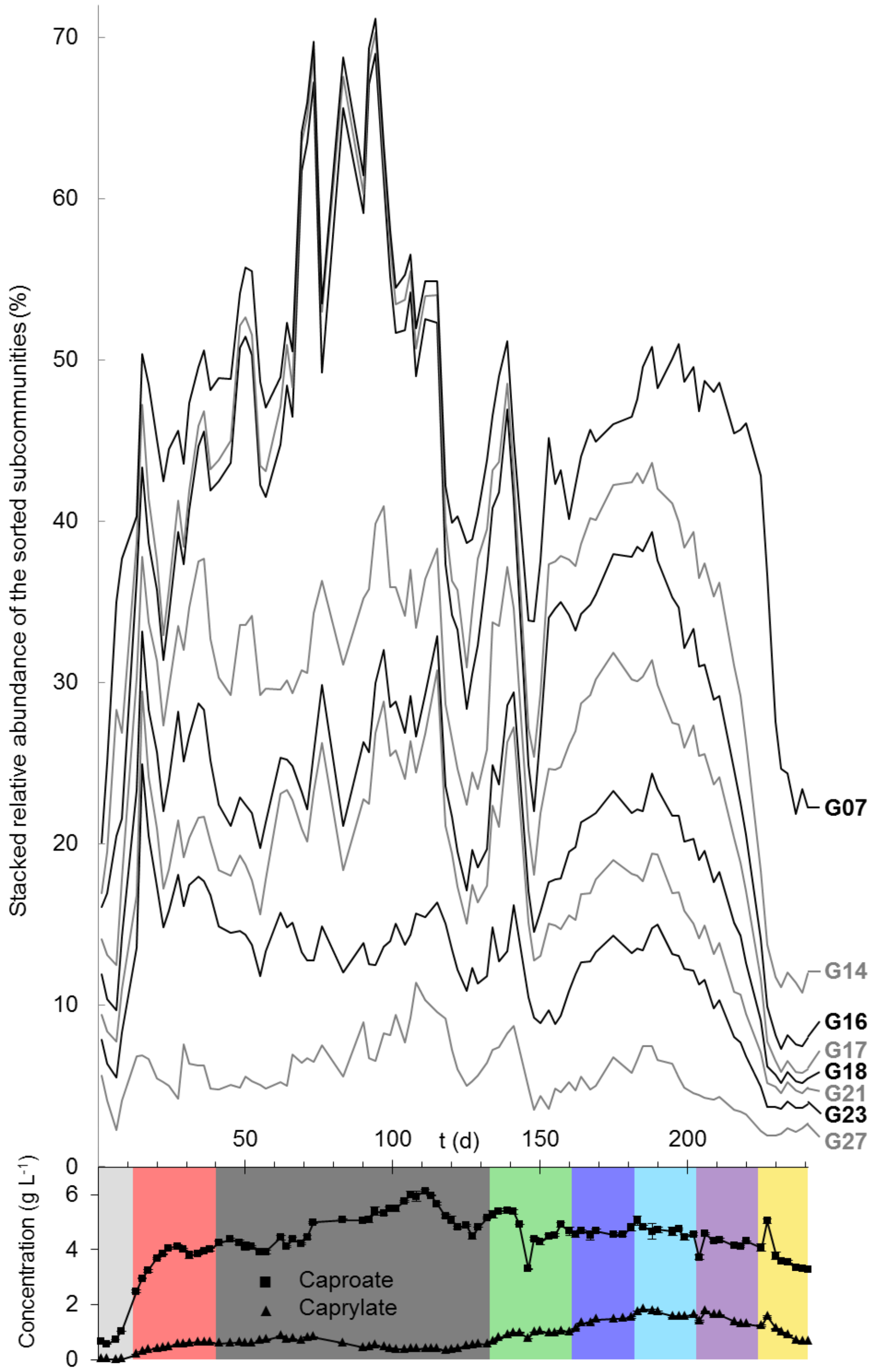

Figure 3 S12: Stacked relative cell abundances of the eight sub-communities of interest, which were sorted and analysed by MiSeq amplicon sequencing over the course of the experiment.

Flow cytometry is able to detect the alternating physiological states of proliferating populations and communities [7,8]. This is elucidated by G16, G17, G18, G21 and G23, which were dominated by the same pair of organisms, namely *Bifidobacterium* and *Olsenella* (Figure 5, main manuscript). Single species can develop multiple distinctly clustering subpopulations comprising cells of different size (linked to FSC) and DNA content (linked to DAPI fluorescence). This effect is generally caused by the proliferation cycle of the respective species, which is in turn tightly connected to its metabolic activity. The respective gates are typically situated close to each other in the gate template and display cells with one, two and multiple chromosomes. This has also been observed in this study (Figure 1 S12) and was clearly exemplified by the *E. coli* BL21 (DE3) strain used as biological control during cytometer set up (Additional file 1 S9).

S13: Miseq amplicon sequencing protocols and details

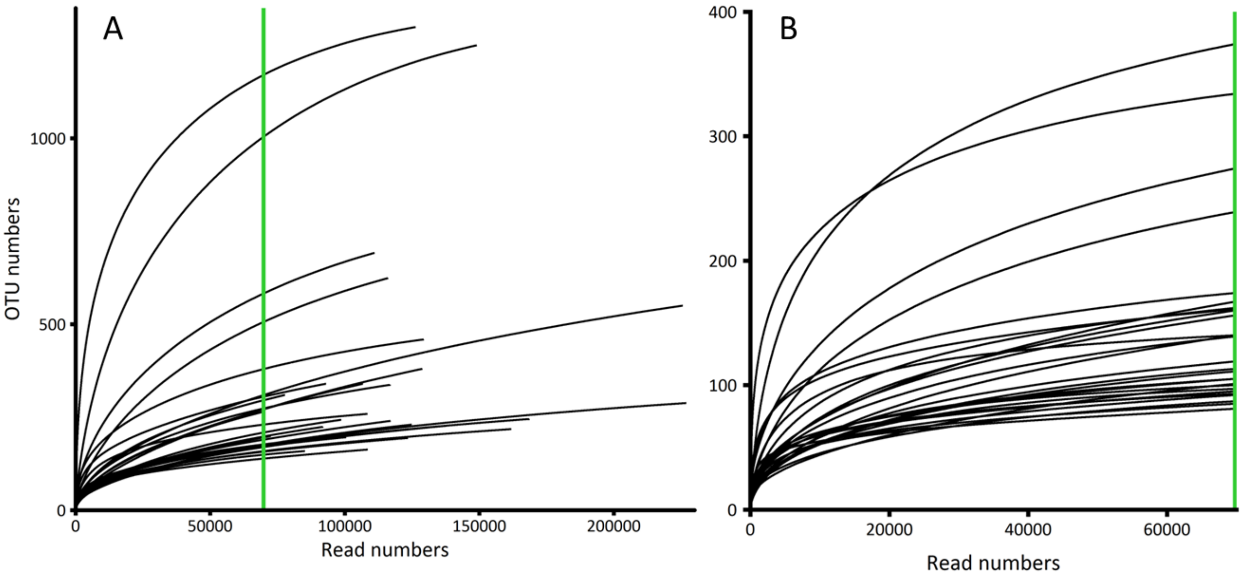

Figure S13: Rarefaction analysis of 16S RNA gene amplicon sequencing reads with **A:** raw data and **B:** data normalised to 69,760 reads (green line) and classified to genus level.

Table S13: Processed 16S rRNA gene sequence specific read numbers of the sorted sub-communities. Normalisation procedure was perfomed with 69,760 reads per sample. Lowest read number was highlighted in red and highest in green.

Sub-community	Day	Read number
G07	24	77841
	220	91814
G14	167	108568
	202	161932
G16	52	72229
	83	85264
G17	52	69760
	185	85607
G18	34	93061
	202	116976
G21	106	108471
	141	106763
G23	52	100467
	185	123433
G27	106	168462
	185	226958

DNA extraction and quality testing

The DNA extraction was performed according to [9]. In short, 70 µL of 10% (wt/vol) Chelex 100 solution (Biorad, Hercules California, USA) was used to extract the DNA from a centrifuged pellet (25 min, 20,000 × g, 4 °C) of 500,000 sorted cells. The samples were heated to 90 °C for 45 min and centrifuged (5 min at 7,000 × g, 4 °C) before 50 µL of the supernatant containing the purified DNA was stored at -20 °C for library preparation. The unsorted whole community samples were resuspended from the dried pellets in 800 µL phosphate bufferd saline (PBS) for 20 min at room temperature. The OD was adjusted to 0.01 (d=5 mm, ʎ=700 nm) in 70 µL of molecular grade water (Qiagen, Hilden, Germany) and the cells were pelleted by centrifugation (25 min, 20,000 × g, 4 °C) to be processed afterwards according to the outlined protocol.

A PCR (polymerase chain reaction) step was performed to evaluate the quality of the isolated DNA by testing their amplified products by gel electrophoresis on a 1.5% agarose gel. The PCR step was done

with 35 cycles in a S1000 Thermal cycler (Biorad, Hercules California, USA) by using the universal primers forward 27F 5'-AGAGTTTGATCMTGGCTCAG-3' and reverse 1492R 5'-TACGGYTACCTTGTTACGACTT-3' according to [10]. All utilised primers were synthesised by Eurofins (Eurofins Scientific, Luxembourg City, Luxembourg).

Mock communitiy

A mock community MBARC26 [11] was used as positive control for the sequencing run as well as the analysis pipeline. MBARC26, composed of 26 cultivable species (23 bacteria, 3 archaea) in different abundances was designed to mimic the diversity of a natural microbial community.

Library preparation for Illumina MiSeq sequencing technique

The V3-V4 region of the bacterial 16S rRNA gene region was amplified with the primers Pro341F 5'-CCTACGGGNBGCASCAG-3' [12] and Pro805R 5'-GACTACNVGGGTATCTAATCC-3' [13]. The PCRs were performed using 20 pmol forward and reverse primers, 4 nmol of dNTP mix (Promega, Fitchburg, Wisconsin, USA), 4 µL 5x Phusion GC solution, 40 nmol of $MgCl_2$ (both provided in the polymerase kit), 0.4 units of Phusion High-Fidelity Polymerase (New England Biolabs, Ipswich, Massachusetts, USA) and 2 µL DNA solution. Nuclease-free water (Qiagen, Hilden, Germany) was added to adjust the final reaction volume to 20 µL. The PCRs were performed with: 3 min of initial denaturation at 95 °C, a denaturation step at 95 °C for 30 s, annealing at 52 °C for 45 s, extension for 45 s at 72 °C and a final extension at 72 °C for 10 min before storage at 4 °C. The primary PCR was run for 20 PCR cycles on sorted samples and 18 cycles on unsorted samples. For each batch, a negative control without DNA was amplified up to 35 cycles and checked via gel electrophoresis (1.5% agarose) to check for contaminations. The amplicons were purified using the Agencourt AMPure XP-Kit (Beckman Coulter, Brea, California, USA) with the recommended protocol. The purified DNA was resuspended in nuclease free water (Qiagen, Hilden, Germany). Two µL of the purified amplicon solution was further amplified by ten secondary PCR cycles using the described parameters and barcoded primers. The purified amplicon fragments were quantified using the Qubit 3.0 (Life technologies, Carlsbad, Califorania, USA) and the HS DNA kit (Life Technologies, Carlsbad, California, USA), and pooled in equimolar amounts. They were analysed at the DSMZ (Deutsche Sammlung von Mikroorganismen und Zellkulturen GmbH, Braunschweig, Germany) using the MiSeq sequencer (Illumina, San Diego, California, USA) with the v3 kit, 2 x 300 bp, 600 cycles. To minimise the technical bias, the PCRs were done in triplicates. The

triplicates were purified and quantified separately. The MBARC26 mock community was processed accordingly to ensure the quality of the PCR steps and the following data analysis.

Sequencing data analysis

The Illumina dataset was quality-trimmed with PRINTSEQ [14] from the 3’ side at a minimum of Q=30 in a window of 20 bases. The remaining sequences were demultiplexed, merged and pre-clustered using Mothur version 1.39 [15]. Chimeras were removed using UCHIME [16]. The OTU classification was done with Mothur’s average neighbour clustering algorithm with a 97% sequence similarity cut off on the SILVA database version 128 [17]. The analysis was based on 3,261,920 forward-reverse overlapped sequences out of 4,146,676 raw sequences. All raw data are available under the BioProject accession number: PRJNA504543.

Rarefraction curves were plotted using ggplot2 package in R [18]. The cleaning procedure yielded a total of 1,883,520 forward-reverse overlapped cleaned sequences. The individual samples were represented with 69,760 (G17 sorted on day 52) to 226,958 (G27 sorted on day 185) cleaned reads. 69,760 reads were chosen as subsampling threshold for the normalisation procedure to allow diversity comparison between samples.

The OTU abundance threshold was set to 0.1%. As the study did not focus on the rare biosphere but the identification of process-relevant active species, the data visualisation focused on organisms displaying over 1% of abundance. 24 out of the 26 species comprising the MBARC26 mock community were recovered. The two species that were not detected in our data set, i.e. *N. dassonvillei* and and *S. bongori*, were of very low abundance in the mock community and also barely detected by [11].

References

1. Popp D, Schrader S, Kleinsteuber S, Harms H, Sträuber H. Biogas production from coumarin-rich plants—inhibition by coumarin and recovery by adaptation of the bacterial community. Stams A, editor. FEMS Microbiol Ecol. 2015;91:fiv103.

2. Urban C, Xu J, Sträuber H, Santos Dantas TR dos, Mühlenberg J, Härtig C, et al. Production of drop-in fuels from biomass at high selectivity by combined microbial and electrochemical conversion. Energy Environ Sci. 2017;10:2231–44.

3. Naumann C, Bassler R, Seibold R, Barth C. Methodenbuch: Die chemische Untersuchung von Futtermitteln. Band III. 1st ed. Speyer: VDLUFA - Verlag; 1997.

4. Van Soest P van, Robertson JB, Lewis BA. Methods for dietary fiber, neutral detergent fiber, and nonstarch polysaccharides in relation to animal nutrition. J Dairy Sci. 1991;74:3583–97.

5. Weißbach F, Strubelt S. Die Korrektur des Trockensubstanzgehaltes von Maissilagen als Substrat für Biogasanlagen. LANDTECHNIK. 2008;2:3.

6. Sträuber H, Bühligen F, Kleinsteuber S, Dittrich-Zechendorf M. Carboxylic acid production from ensiled crops in anaerobic solid-state fermentation - trace elements as pH controlling agents support microbial chain elongation with lactic acid. Eng Life Sci. 2018;0:1–12.

7. Müller S. Modes of cytometric bacterial DNA pattern: a tool for pursuing growth. Cell Prolif. 2007;40:621–39.

8. Müller S, Vogt C, Laube M, Harms H, Kleinsteuber S. Community dynamics within a bacterial consortium during growth on toluene under sulfate-reducing conditions. FEMS Microbiol Ecol. 2009;70:586–96.

9. Koch C, Günther S, Desta AF, Hübschmann T, Müller S. Cytometric fingerprinting for analyzing microbial intracommunity structure variation and identifying subcommunity function. Nat Protoc. 2013;8:190–202.

10. Lane DJ. 16S/23S rRNA Sequencing. Nucleic Acid Tech Bact Syst. Chichester, United Kingdom: John Wiley and Sons; 1991. p. 115–75.

11. Singer E, Andreopoulos B, Bowers RM, Lee J, Deshpande S, Chiniquy J, et al. Next generation sequencing data of a defined microbial mock community. Sci Data. 2016;3:160081.

12. Takahashi S, Tomita J, Nishioka K, Hisada T, Nishijima M. Development of a Prokaryotic Universal Primer for Simultaneous Analysis of Bacteria and Archaea Using Next-Generation Sequencing. Bourtzis K, editor. PLoS ONE. 2014;9:e105592.

13. Herlemann DP, Labrenz M, Jürgens K, Bertilsson S, Waniek JJ, Andersson AF. Transitions in bacterial communities along the 2000 km salinity gradient of the Baltic Sea. ISME J. 2011;5:1571–9.

14. Schmieder R, Edwards R. Quality control and preprocessing of metagenomic datasets. Bioinformatics. 2011;27:863–4.

15. Schloss PD, Westcott SL, Ryabin T, Hall JR, Hartmann M, Hollister EB, et al. Introducing mothur: Open-Source, Platform-Independent, Community-Supported Software for Describing and Comparing Microbial Communities. Appl Environ Microbiol. 2009;75:7537–41.

16. Edgar RC, Haas BJ, Clemente JC, Quince C, Knight R. UCHIME improves sensitivity and speed of chimera detection. Bioinformatics. 2011;27:2194–200.

17. Quast C, Pruesse E, Yilmaz P, Gerken J, Schweer T, Yarza P, et al. The SILVA ribosomal RNA gene database project: improved data processing and web-based tools. Nucleic Acids Res. 2012;41:D590–6.

18. Wickham H. ggplot2: Elegant Graphics for Data Analysis. 2nd ed. New York: Springer-Verlag; 2009.
