# Supplementary figures and images for: Key sub-community dynamics of medium-chain carboxylate production
Source: Microb Cell Fact. 2019 May 28;18:92. doi: 10.1186/s12934-019-1143-8 (PMC6537167; doi:10.1186/s12934-019-1143-8)

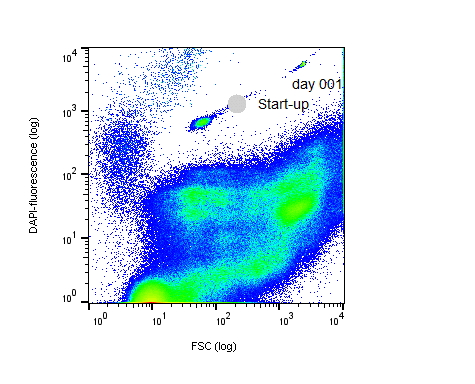

Supplement: Supplementary file 2 — Additional file 2. Additional information containing a short movie in a.gif file with the cytometric fingerprints of all 89 time points as 500 ms frames. [file 12934_2019_1143_MOESM2_ESM.gif]
